# Intratumoural plasma cells mediate a pro-inflammatory response to kinase inhibition and successful immune checkpoint blockade therapy in malignant peripheral nerve sheath tumours

**DOI:** 10.1016/j.ebiom.2026.106413

**Published:** 2026-08-01

**Authors:** Joshua J. Lingo, Ryan Reis, Altay Koyas, Ellen Voigt, Zeb R. Zacharias, Chantal Allamargot, Emma L. Hornick, Juan A. Raygoza Garay, Courtney A. Kaemmer, Elizabeth C. Elias, Ali Jabbari, Connor R. Wilhelm, Alexander W. Boyden, Nitin J. Karandikar, Patrick Breheny, David K. Meyerholz, Rebecca D. Dodd, Jon C.D. Houtman, Benjamin W. Darbro, Dawn E. Quelle

**Affiliations:** a Cancer Biology Graduate Program, University of Iowa, Iowa City, IA, USA; b The Department of Neuroscience and Pharmacology, University of Iowa, Iowa City, IA, USA; c Medical Scientist Training Program, University of Iowa, Iowa City, IA, USA; d Translation Immunology Core, University of Iowa, Iowa City, IA, USA; e Central Microscopy Core Research Facility, University of Iowa, Iowa City, IA, USA; f Holden Comprehensive Cancer Center, University of Iowa, Iowa City, IA, USA; g Maximizing Access to Research Careers (MARC) Program, University of Iowa, Iowa City, IA, USA; h The Department of Dermatology, University of Iowa, Iowa City, IA, USA; i The Department of Pathology, University of Iowa, Iowa City, IA, USA; j The Department of Biostatistics, University of Iowa, Iowa City, IA, USA; k The Department of Internal Medicine, University of Iowa, Iowa City, IA, USA; l The Department of Microbiology and Immunology, University of Iowa, Iowa City, IA, USA; m The Department of Pediatrics, University of Iowa, Iowa City, IA, USA

**Keywords:** MPNST, Plasma cells, PD-L1, CD8+ T cells, NK cells, Immune checkpoint blockade (ICB)

## Abstract

**Background:**

The role of intratumoural plasma cells in immune checkpoint blockade (ICB) therapy has never been tested although their presence is linked with improved response and survival in people with cancer. Malignant peripheral nerve sheath tumours (MPNSTs) are deadly sarcomas with minimal responsiveness to ICB therapies. Strikingly, drugs inhibiting cyclin-dependent kinases 4/6 (CDK4/6) and MEK sensitise *de novo* MPNSTs to immunotherapy targeting programmed death-ligand 1 (PD-L1), which correlates with increased intratumoural plasma cells. Here, we tested if plasma cells mediate MPNST response to anti-PD-L1 therapy and how they modulate tumour immune responsiveness to CDK4/6-MEK inhibition.

**Methods:**

Anti-tumour activity of PD-L1 inhibition, with or without CDK4/6-MEK inhibition, was measured in *de novo* MPNSTs within wild-type versus plasma cell-deficient mice. Plasma cell-dependent effects of CDK4/6-MEK inhibition on priming the MPNST immune environment were determined by single cell transcriptomics and immune cell analyses.

**Findings:**

Plasma cell-deficient MPNSTs failed to respond to anti-PD-L1 monotherapy and were no longer sensitised by CDK4/6-MEK inhibition to immunotherapy. Following kinase inhibitor treatment, plasma cells were necessary for a pro-inflammatory response marked by increased tumour infiltration and activation of natural killer (NK) and CD8+ T cells, major histocompatibility class I antigen presentation, and decreased M2 macrophages. By comparison, elevated CD4+ T cell and B cell infiltration dominated the plasma cell knockout phenotype.

**Interpretation:**

Plasma cells favourably remodel the tumour immune environment for anti-tumour immunity and are critical for successful ICB therapy in MPNSTs. These findings may inform ICB treatment strategies and patient stratification for therapy of many tumour types.

**Funding:**

This research was supported by University of Iowa Sarcoma Research Program awards, the 10.13039/100016618Gilbert Family Foundation, and 10.13039/100000002NIH grants T34-GM141143, T32-GM067795, F31-CA281312, P30-CA086862, and R01-NS119322.

**Research in context**

**Evidence before this study**

For many types of cancer, intratumoural plasma cells have been *correlated* with better patient survival and improved response to immune checkpoint blockade (ICB) therapies. However, the biology underlying those associations *is not understood* and no study has examined the requirement of plasma cells in immunotherapy response. Compelling data in malignant peripheral nerve sheath tumours (MPNSTs) showed that dual kinase inhibition of oncogenic CDK4/6 and MEK induced intratumoural plasma cell accumulation and sensitised tumours to ICB therapy. While CDK4/6-MEK inhibition is known to enhance anti-tumour immunity in other tumour types by activating CD8+ T cells and/or natural killer (NK) cells, a role for plasma cells has never been explored.

**Added value of this study**

Studies were performed in MPNSTs, an under-researched cancer that normally responds poorly to ICB monotherapies. This investigation shows that intratumoural plasma cells are essential for successful ICB therapy and mediating a pro-inflammatory response to CDK4/6-MEK inhibition. Specifically, plasma cells support anti-tumour immunity by remodelling both adaptive (CD8+ T cells up and CD4+ T cells down) and innate (NK cells up and M2 macrophages down) components of the tumour immune milieu. Findings reveal that known anti-tumour, immunomodulatory effects of CDK4/6-MEK inhibitor therapies are dependent on intratumoural plasma cells.

**Implications of all the available evidence**

The fundamental advance in understanding how plasma cells promote successful ICB immunotherapy is likely applicable to other solid tumours and may guide new therapeutic strategies in which plasma cell-inducing agents are combined with ICB antibodies. Moreover, an increased presence of intratumoural plasma cells in tumour specimens may streamline clinical decisions regarding which patients are most likely to benefit from ICB therapy.

## Introduction

Malignant Peripheral Nerve Sheath Tumours (MPNSTs) are an aggressive subtype of sarcoma that originates from the myelinating nerve sheath. MPNSTs can arise sporadically or in patients with Neurofibromatosis Type 1 (NF1), the most common, heritable tumour predisposition syndrome worldwide.^1^^,^^2^ Unfortunately, MPNSTs are notoriously unresponsive to chemotherapy, lack approved targeted therapies, and generally are insensitive to immune checkpoint blockade (ICB) therapy. The latter is thought to reflect an immunologically “cold” nature of MPNSTs although it is an understudied area in MPNST research.^3^

MPNSTs are driven by hyperactivated Ras/MEK/ERK signalling initiated by loss of *NF1*, a tumour suppressor gene that encodes neurofibromin, a Ras GTPase activating protein (GAP) that converts Ras into its inactive, GDP-bound form.^4^ In individuals with NF1, mutation of the *NF1* gene promotes formation of benign plexiform neurofibromas (PNFs), precursor lesions that can transform into MPNSTs in up to one-third of patients.^2^ Additional alterations cooperate with *NF1* loss to support PNF transition into MPNSTs.^2^^,^^5^, ^6^, ^7^ Two of the most common changes driving that process are loss of *INK4a* and *ARF* (collectively called *CDKN2A*)^7^ and overexpression of RABL6A,^8^ events that converge functionally to activate oncogenic cyclin-dependent kinases 4 and 6 (CDK4/6) and turn off the Retinoblastoma 1 (RB1) tumour suppressor.^9^^,^^10^ Elevated Ras/MEK activity also upregulates CDK4/6 through MYC-mediated transcription of *CDK4, CDK6,* and *CCND1* (which encodes the cyclin D1 regulatory subunit required for CDK4/6 activity).^11^^,^^12^ Efforts targeting MEK or CDK4/6 individually in preclinical MPNST models have yielded unsatisfactory results due to rapid resistance,^13^, ^14^, ^15^, ^16^, ^17^, ^18^, ^19^ prompting exploration of combination therapies targeting those factors in MPNSTs.

Preclinical studies pairing CDK4/6 and MEK inhibitors have proven effective at suppressing Ras pathway-driven cancers through activation of anti-tumour immunity. This includes MPNSTs,^20^ ovarian cancer,^21^ colorectal cancer,^22^ melanoma,^23^^,^^24^ as well as adenocarcinomas of the lung^25^ and pancreas.^26^ In *de novo* MPNSTs induced by *Nf1/Ink4a/Arf* inactivation in the mouse sciatic nerve, dual CDK4/6-MEK inhibition provokes an anti-tumour immune response featuring activated CD8+ T cells and intratumoural plasma cell accumulation.^20^ The plasma cell phenotype, the first to be reported for this drug combination in any tumour, is compelling because intratumoural plasma cells predict prolonged patient survival^27^, ^28^, ^29^, ^30^ and improved response to ICBs^28^^,^^31^, ^32^, ^33^, ^34^ for most human cancers. Indeed, combining CDK4/6 and MEK inhibition with ICB therapy targeting programmed death ligand 1 (PD-L1) causes sustained MPNST regression and extended mouse survival with some mice exhibiting cure.^20^ Synergistic tumour killing by combining CDK4/6 and MEK inhibitors with another ICB agent, anti-PD-1 antibodies, has been achieved in other preclinical tumour models.^21^^,^^25^^,^^26^ However, no study has investigated the requirement of plasma cells for the anti-tumour immune response to ICB therapy or determined how they influence the tumour immune milieu following CDK4/6-MEK inhibition.

Here, we directly test the role of plasma cells in the response to CDK4/6-MEK inhibition and/or anti-PD-L1 therapy in *de novo* MPNSTs within mice that either contain or lack plasma cells. Our findings demonstrate that plasma cells are critical for successful ICB therapy and mediate a pro-inflammatory state following CDK4/6-MEK inhibition characterised by favourable adaptive (increased CD8+ T cell) and innate (increased NK cell, reduced M2 macrophage) immune cell changes. Understanding the unique role of plasma cells in controlling therapy-induced, anti-tumour immunity may have broad relevance to the effective treatment of solid cancers with immune-based therapies.

## Methods

### Sex as a biological variable

Our study examined male and female animals, and similar findings are reported for both sexes.

### Primary MPNST generation

Mice were housed with a 12-h light-dark cycle and free access to food and water. All mouse handling was conducted in compliance with the Institutional Animal Care and Use Committee (IACUC) policies at the University of Iowa. Mice were housed in barrier conditions and do not develop systemic or opportunistic infections. These requirements adhere to the NIH Guide for the Care and Use of Laboratory Animals and the Public Health Service Policy on the Humane Care and Use of Laboratory Animals. All efforts were made to minimise animal suffering. Primary MPNSTs were generated via adenoviral delivery of Cas9 and sgRNAs targeting *Nf1* and *Cdkn2a* to the left sciatic nerve as previously reported.^35^ Tumours were initiated in wild-type C57BL/6N (n = 53) and plasma cell-knockout (PC-ko, *AID*−/−; *μS*−/−, Activation-induced cytidine deaminase (AID)/secretory mu-chain (μS) double knockout) mice on C57BL6/J background (n = 46).^36^ Male and female mice were used equally.

### Flow cytometric validation of *AID*−/−; *μS*−/− mice

Fresh splenocytes were harvested from wild-type and PC-ko mice via physical disruption of the spleen and then passed through a 70 μM strainer.^37^ Single cell suspensions of splenocytes were stained for viability with Zombie NIR (Biolegend, cat. 423105) and Fc receptors were blocked with anti-CD16/32 (Biolegend, clone 93) for 10 min on ice in the dark. Samples were washed in Cell Staining Buffer (Biolegend, 420201) and incubated with the following antibodies for 30 min on ice: CD45 (clone 30-F11, BV510, Biolegend #103137, RRID: AB_2561392), B220 (clone RA3-RB2, APC/Fire 810, Biolegend #103277, RRID: AB_2860603), CD3 (clone 17A2, Spark NIR 685, Biolegend #100261, RRID: AB_2832259), and CD138 (clone 281-2, APC, Biolegend #142505, RRID: AB_10960141). Splenocytes were washed via centrifugation, fixed in FluoroFix Buffer (Biolegend, cat. 422101), washed again, and stored overnight at 4 °C. Samples were analised on the Cytek Aurora. Flow cytometric analyses were performed using FlowJo 10.9.0 (Becton, Dickson and Company). Plasma cells were identified as live, CD45+CD3−B220−CD138+ singlets. Fluorescence minus one (FMO) controls were used to identify positive and negative populations.

### *In vivo* tumour growth analyses and drug treatment conditions

Tumour size was measured with callipers and tumour volume was calculated using the formula (length x width x thickness x π)/6. After tumour detection (∼250 mm^3^), mice were randomly enrolled into treatment groups. Daily, mice received, by oral gavage, either a vehicle control (50 mM sodium-L-lactate, pH 4.0 plus 1% DMSO) or kinase inhibitors (100 mg/kg palbociclib [CDK4/6 inhibitor] plus 1 mg/kg mirdametinib [MEK inhibitor]). For the first three weeks of therapy, starting upon tumour detection, mice received IgG control (10 mg/kg anti-keyhole limpet haemocyanin [BioXCell BE0090, RRID: AB_1107780] diluted in InVivoPure pH 7 [BioXCell IP0070]) or anti-PD-L1 (10 mg/kg anti-PD-L1 [BioXCell BE0101, RRID: AB_10949073] diluted in InVivo Pure pH 6.5 [BioXCell IP0065]) intraperitoneally, twice per week for a total of 6 doses. Each mouse included in the study was euthanised due to tumour burden at a preset endpoint of ∼1250 mm^3^. Tumours were immediately harvested and sectioned for RNA/protein analysis (flash-frozen) and histopathological analysis (formalin-fixed paraffin-embedded, FFPE).

### Preparation of *de novo* MPNSTs for single cell RNA sequencing

After tumour detection (∼450 mm^3^), wild-type C57BL/6N and *AID*−/−; *μS*−/− mice received 100 mg/kg palbociclib plus 1 mg/kg mirdametinib daily by oral gavage until they regressed to ∼250 mm^3^, which occurred within 4–6 days. Upon reaching the desired endpoint, mice were euthanised and tumours were immediately excised. Each tumour was divided into sections for flash freezing, immunohistochemistry (FFPE), or digested into single cell suspensions for single cell RNA sequencing (scRNA seq). To develop single cell suspension, small portions of each tumour were cut into small cubes (∼8 mm^3^). Minced tumours were then dissociated using the Mouse Tumour Dissociation Kit (Miltenyi Biotec 130-096-730) with all dissociation steps requiring media performed in DMEM. Tumour dissociation was completed using the gentleMACS Dissociator (Miltenyi Biotec cat. #130-093-235). After mechanical dissociation, samples were enzymatically digested according to manufacturer protocols on an orbital shaker at 250 rpm at 37 °C for 45 min. Suspensions were filtered using 70 μM mesh strainer and percent viability determined via trypan blue exclusion. Viable suspensions were cryopreserved in 90% foetal bovine serum (FBS) and 10% DMSO at a concentration of 1 × 10^6^ cells/mL. Cryopreserved single cell suspensions of *de novo* MPNSTs were thawed at 37 °C on the day of sequencing and viability reconfirmed via trypan blue exclusion. Only single cell suspensions with viability greater than 80% were included in GEM preparation using the 10X Genomics Chromium controller at the Iowa Institute of Human Genetics Genomic Division. cDNA libraries were generated using the Chromium Single Cell 3’ v3.1 kit. Sequencing was conducted on the Illumina NovaSeq 6000 using an S1 flow cell.

### scRNA sequencing analyses

FASTQ files were concatenated across sequencing lanes and aligned to the GRCm39-2024-A mouse reference genome using cellranger (v7.0.1) with default settings. Ambient RNA was removed from each raw_feature_bc_matrix.h5 file via cellbender (v0.3.0), using default parameters except for the following: epochs = 50; this choice was made following inspecting of the ELBO convergence plots for each sample. The resulting filtered.h5 outputs were loaded into Rstudio (R v4.4.1) and converted into Seurat objects using the Seurat (v5.1.0) package with min.cells = 5 and min.features = 300. Percent mitochondrial and ribosomal reads were calculated for each cell and doublets called using scDblFinder (v1.18.0). One sample (B_009, WT) was excluded due to what appeared to be persistent RNA contamination (ambient RNA), and the remaining samples were merged into a single Seurat object. Additional filtering was then applied via the following: db.class = = “singlet” & nFeature_RNA > 250 & nCount_RNA > 400 & nCount_RNA < 150,000 & percent.mt < 25 & percent.rb < 40. The filtered dataset underwent normalisation with SCTransform, regressing out percent.mt and nCount_RNA. Principal component analysis was performed on the SCT assay, followed by harmony (v1.2.0) integration to correct for batch effects. Uniform Manifold Approximation and Projection (UMAP) embeddings were computed on the harmony-corrected dimensions, and graph-based clustering was completed using the first 20 dimensions. Marker genes were identified with the Seurat FindAllMarkers function, and major cell-type identities were assigned based on these results.

CellChat (v2.1.2) was used to identify differences in cell–cell communication between the genotypes of interest. CellChat objects were generated separately for each genotype from their SCT assays, merged into a combined CellChat object, and a comparative analysis was performed, comparing the PC_KO to the WT genotype results. Metaprograms (MPs) were identified using GeneNMF (v0.6.0). MPNST cells were subset and split by sample. The SCT assay was used as input to multiNMF, with parameters k = 6:8, L1 = c (0,0), scale = TRUE, centre = TRUE, and nfeatures = 1000. Eight consensus MPs were identified via getMetaPrograms (max.genes = 50) and visualised in a heatmap of overlapping genes. Individual cells were scored for expression of each MP using AddModuleScore_UCell. Gene ontology was performed for each MP using clusterProfiler. For major cell types of interest, these cells were subset from the original object and re-processed individually to create celltype-specific pca and umap reduced dimensions and clusters. Marker genes were again identified with FindAllMarkers, and the clusters were labelled according to these differentially expressed genes (DEGs). DEGs were also identified as a function of genotype for genotype-specific gene onotology. Heatmaps were generated via the R2easyviz (v0.1.0) package. Cells were down sampled and grouped according to their subcluster and the expression of the top DEGs, as a function of percent difference in expression (pct.1 - pct.2), for each was scaled and centered for visualisation. Stacked bar charts were generated for each subcluster of interest and coloured by genotype. The proportion of cells from each genotype was calculated as a function of total cells within each cluster and presented as percent of total. Select feature plots and dot plots were generated using the Seurat package.

### Mouse tumour RNA isolation and quantitative RT-PCR analyses

RNA was isolated from flash frozen mouse tumours using Qiagen RNAeasy Mini Plus Kit. RNA yield and purity were obtained using the Trinean DropSense 16. cDNA was synthesised from 200 ng total RNA using SuperScriptTM III First-Strand Synthesis System (Invitrogen, cat. 18080051). Diluted cDNA was used for real-time PCR (cycle conditions: denaturation at 95 °C for 10 min, followed by 40 cycles of 95 °C for 15 s and 45–50 °C for 1 min), using SYBR Green for detection (Bio Rad, cat. 1708880). Gene-specific primers were used to determine the fold change in mRNA expression using the 2^−ΔΔCt^ method, calibrated to *Gapdh* mRNA expression. Primer sequences are in Supplementary Table S1.

### Histopathological analysis of mouse MPNSTs

Upon euthanasia, tumours were harvested and stored in 10% neutral-buffered formalin. Tumours were embedded in paraffin, and sectioned (5 μm) onto glass slides, and hydrated. Heat-induced epitope retrieval with Citrate-based Antigen Unmasking Solution pH 6.0 (Vector Laboratories H-3300-250) at greater than 95 °C for 5 min followed by 5 min of rest. This was repeated once. After blocking, tissue sections were immunostained with the antibodies in Supplementary Table S2 in blocking buffer (5% Normal Donkey [Sigma, D9663]/Goat Serum [Sigma, G-9023] in PBS) or Vector M.O.M. Immunodetection kit (Vector Laboratories, BMK-2202) overnight at 4 °C. Sections were then washed in PBS and incubated with goat anti-rabbit IgG F (ab’)_2_ conjugated to Alexa-Fluor 488, 568, or 647 for 1 h at room temperature. All secondary antibodies were used at a 1:500 dilution in blocking buffer. Antibodies that required signal amplification (SA) for visualisation were incubated with biotinylated goat antibodies raised against the host species of the primary antibody for 1 h followed by a PBS wash and incubation with streptavidin-Alexa Fluor 647 conjugate for 1 h. Before any work with biotinylated antibodies, endogenous biotin was blocked with egg substitute solution and fat-free milk for 15 min each. After all antibody incubations, slides were washed with PBS and glass coverslips were mounted using Prolong Glass Antifade Mountant with NucBlue (ThermoFisher P36981). Sections of tissue were examined by at least two observers (including a pathologist) and morphometric analysis was performed using the post-examination method of masking to group assignment.^38^ At least 4 high-power field images (HPF, 40X) were obtained for each tumour section and stain using a Zeiss LSM 980. Positive cells per HPF were counted manually and fluorescent intensity (relative fluorescent units, RFU) was quantified using Fiji/ImageJ. Lymphoid infiltration was quantified from haematoxylin and eosin-stained tissue sections. Percent lymphoid area was defined as the total area of lymphoid infiltration divided by the total area of the tumour section.

### Flow cytometric analyses of tumour infiltrating immune cells

Following tumour collection and enzymatic dissociation using the Mouse Tumour Dissociation Kit (Miltenyi Biotec 130-096-730), samples were filtered using 70 μm nylon mesh prior to staining. Single-cell suspensions were stained using a modified version of a 40-colour full immune spectral flow cytometry panel that has been previously described.^39^ Briefly, cells were plated in a 96-well round-bottom polystyrene plate and washed with PBS. Cells were then treated with Live/Dead Blue (Invitrogen) in PBS for 15 min at room temperature. Following Live/Dead stain, cells were pre-blocked in 50 μL of FACS buffer (PBS w/5% FBS and 0.01% sodium azide) supplemented with CellBlox™ (Invitrogen), True-Stain Monocyte Blocker™, 2% Rat serum, 2% Hamster serum, and 2% Mouse serum for 15 min at room temperature. Cells were then stained with an antibody master mix containing the antibodies listed in Supplementary Table S3 for 30 min at room temperature protected from light. Cells were then washed twice with FACS buffer and then fixed with BD FACS Lysis Buffer for 10 min at room temperature, according to the manufacturer (Waters Biosciences). Following fixation, cells were washed twice with PBS and then stored in PBS at 4 °C protected from light until analysis. Data were collected using a 5-laser Cytek Aurora and data were analysed with SpectroFlo (Cytek) and FlowJo (Waters Biosciences) software using the gating strategy shown in Supplementary Fig. S1.

### Analysis of TCGA bulk RNA sequencing

Immune cell prediction was performed on publicly available RNA sequencing data from TCGA portal (SARC cohort). CIBERSORT was run using the LM22 signature matrix file, in relative (not absolute) mode, with quantile normalisation disabled, and 1000 permutations. Intercellular correlations were determined the Pearson correlation method. High and low cell fractions were determined by the upper and lower 45th percentile, respectively, after the exclusion of any samples with null values. Kaplan–Meier analysis was performed to determine statistical differences in cohort survival.

### Statistics

Quantified data are presented as the mean and all error bars are SEM. P values, unless otherwise specified, were obtained by One-way ANOVA and adjusted for multiple comparisons using the indicated method in GraphPad Prism 10 (GraphPad Software). P values less than 0.05 were considered statistically significant. Statistical analysis of median survival was performed using Kaplan–Meier Analysis. “Cured” tumour-bearing mice were excluded from statistical analyses regarding tumour growth and regression.

### Study approval

All mouse studies adhered to protocols approved by the Institutional Animal Care and Use Committees at the University of Iowa (Protocol 3092074: Targeting RB1 Pathway in Sarcomas). All efforts were made to minimise animal suffering.

### Role of funders

The study sponsors had no role in study design, the collection, analysis, and interpretation of data, writing of the report, or the decision to submit the paper for publication.

## Results

### Plasma cells are essential for MPNST response to anti-PD-L1 therapy

It is increasingly appreciated that the presence of B cells and plasma cells in human tumours correlates with improved response to ICB therapy,^28^^,^^31^, ^32^, ^33^, ^34^ although their exact role in anti-tumour immunity is poorly understood. Preclinical studies in melanoma and ovarian cancer showed that B cells are required for response to ICB therapy alone or combined with other therapies, including CDK4/6 inhibitors.^21^^,^^40^^,^^41^ No study to date, however, has investigated the role of plasma cells in ICB therapy response.

**Fig. 1:**
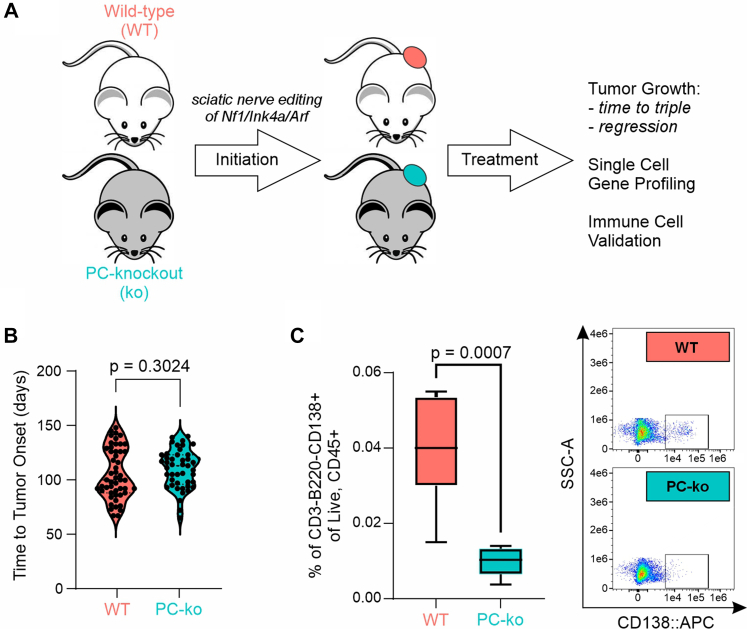
**Loss of plasma cells does not alter the initiation of *de novo* MPNSTs generated by inactivation of *Nf1* and *Cdkn2a*.** (A) Schematic depicting generation of *de novo* MPNSTs via CRISPR-mediated inactivation of *Nf1* and *Cdkn2a* (encoding *Ink4a* and *Arf*) in the sciatic nerve of wild-type (WT) and AID/μs−/− mice (PC-knockout, PC-ko). (B) Quantification and comparison of time to tumour onset of all MPNSTs in this study. (C) Population frequencies of plasma cells relative to all Live, CD45+ cells, with representative pseudocolour plots on the right. Plasma cells were defined as CD3−B220−CD138+ cells. P values were obtained using a Student’s t test.

Given our recent findings that dual CDK4/6-MEK inhibition in *de novo* MPNSTs synergistically increases intratumoural plasma cells (not B cells) and sensitises tumours to anti-PD-L1 therapy,^20^ we sought to test the requirement of plasma cells for ICB response in MPNSTs. To do so, *de novo* MPNSTs were generated in wild-type (WT) mice as well as *AID−/−; μS−/−* knockout mice devoid of plasma cells. The loss of activation-induced cytidine deaminase (*AID*) and the secretory mu-chain (*μS*) genes (hereafter termed PC-ko for plasma cell knockout) yields agammaglobulinemic mice that retain B cells and plasmablasts but selectively lack plasma cells.^36^ The T cell compartment in PC-ko mice is intact.^36^ Using established methods,^8^^,^^20^^,^^35^^,^^42^ *de novo* MPNSTs were induced by CRISPR-Cas9 editing of endogenous *Nf1* and *Cdkn2a* (encodes both *Ink4a* and *Arf*) in the sciatic nerve of wild-type and PC-ko mice (Fig. 1A). MPNSTs in PC-ko mice initiated similarly to those in wild-type mice with no difference in tumour onset (Fig. 1B). The loss of plasma cells in PC-ko mice was validated by flow cytometric identification of live splenic CD45+B220−CD138+ cells^36^ (Fig. 1C). These data show that plasma cells do not influence MPNST initiation.

**Fig. 2:**
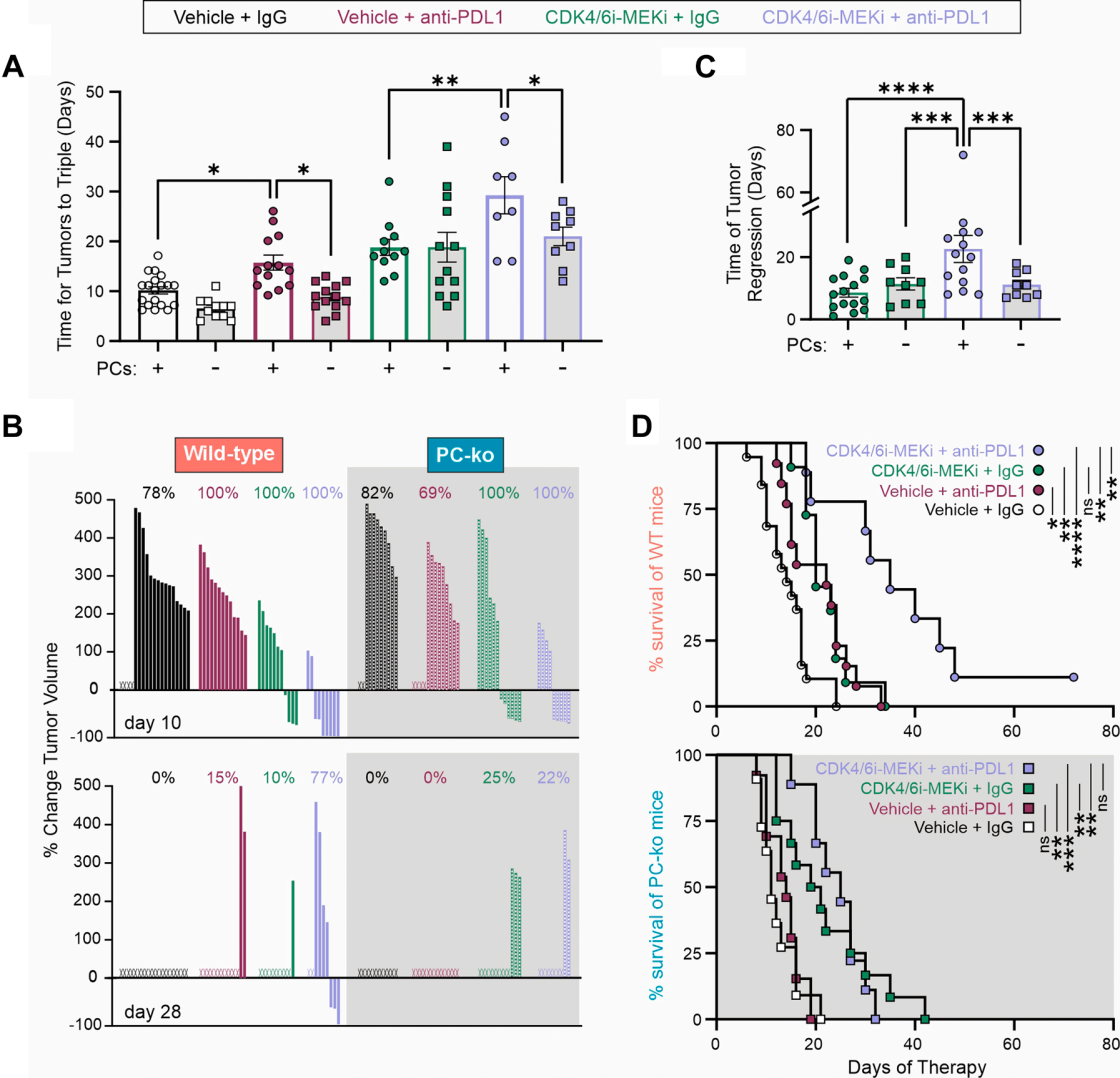
**Plasma cells are required for successful response to anti-PD-L1 therapy in *de novo* MPSNTs.** Wild-type and PC-ko mice bearing *de novo* MPNSTs, initiated by *Nf1/Ink4a/Arf* editing in the sciatic nerve, were treated daily with vehicle or CDK4/6-MEK inhibitors (palbociclib at 100 mg/kg, mirdametinib at 1 mg/kg). Mice also received 2 weekly i.p., injections of IgG control or anti-PD-L1 antibodies for the first 3 weeks of therapy. (A) Time (in days) it took for MPNSTs to triple in volume. (B) Waterfall plots showing percent change in tumour volume at days 10 (top) and 28 (bottom) of treatment. (C) Time (in days) that tumours treated with CDK4/6 and MEK inhibitors ± anti-PD-L1 therapy regressed, as defined by the measured tumour volume being less than volume at detection. (D) Survival of wild-type (WT, top) and plasma cell-deficient (PC-ko, bottom) mice bearing MPNSTs treated with the indicated therapies. Error bars are SEM. P value was obtained via two-way ANOVA (panels A and C) or through Kaplan–Meier analysis (panel D). ns, not significant. In panels A and C, only statistically significant comparisons are shown (∗, P < 0.05, ∗∗, P, <0.01, ∗∗∗, P < 0.001, ∗∗∗∗, P < 0.0001).

To directly determine the necessity of plasma cells for tumour response to ICB therapy, *de novo* MPNSTs in wild-type and PC-ko mice were treated with anti-PD-L1 antibodies alone, inhibitors to CDK4/6 (palbociclib) plus MEK (mirdametinib), or the triple combination versus vehicle with IgG control. Therapy began once tumours reached approximately 250–350 mm^3^ in size. Tumours in wild-type mice responded to the treatments in an identical manner to our previous study.^20^ Control vehicle/IgG-treated tumours tripled in volume at similar rates in wild-type and PC-ko mice (Fig. 2A). Likewise, in both genotypes, treatment with CDK4/6-MEK inhibitors similarly delayed MPNST growth relative to controls. Differences in tumour progression between wild-type and PC-ko mice were only seen following treatment with anti-PD-L1 therapy, either alone (vehicle + anti-PDL1) or when combined with CDK4/6-MEK inhibition (CDK4/6i-MEKi + anti-PDL1). Under those conditions, the slower tumour growth caused by anti-PD-L1 therapy in wild-type animals was completely lost in PC-ko mice (Fig. 2A). Thus, plasma cells are required for MPNST response to ICB therapy.

The above results corresponded with more pronounced tumour regression in plasma cell-containing MPNSTs treated with CDK4/6-MEK-PDL1 targeted therapy. A greater magnitude of tumour shrinkage (seen by the negative fold change in tumour volume) was sustained over time for CDK4/6-MEK-PDL1 targeted MPNSTs in wild-type mice, but this was lost in PC-ko mice (Fig. 2B). Indeed, wild-type tumours treated with CDK4/6-MEK-PDL1 inhibitor therapy had a >2-fold longer average time of tumour regression relative to identically treated PC-ko tumours, which instead matched the shorter time of tumour regression caused by CDK4/6-MEK inhibitors alone in wild-type and PC-ko (Fig. 2C). These data demonstrate that MPNSTs lacking plasma cells are no longer sensitised by CDK4/6-MEK inhibition to undergo extended tumour regression in response to anti-PD-L1 therapy.

The inability of ICB therapy to suppress MPNST growth in the absence of plasma cells directly correlated with reduced survival of those mice. Differences in the response to ICB therapy between wild-type and PC-ko mice were seen as early as day 10 post-therapy where only 69% of PC-ko mice treated with anti-PD-L1 alone were alive versus 100% of wild-type mice (Fig. 2B, top). At day 28, no PC-ko mice receiving PD-L1 monotherapy were alive compared to 15% of wild-type mice (Fig. 2B, bottom). Likewise, for treatment with CDK4/6-MEK inhibitors plus anti-PD-L1 therapy, only 22% of PC-ko mice were surviving at day 28 with no tumours regressing whereas 77% of wild-type mice were alive with 3 of 9 tumours still regressing (Fig. 2B, bottom). Kaplan–Meier analysis similarly revealed that loss of plasma cells (bottom graph) abolished the extended survival and ∼10% cure rate obtained with anti-PD-L1 plus CDK4/6-MEK inhibition in wild-type mice^20^ (Fig. 2D). Moreover, while survival curves for anti-PD-L1 monotherapy and CDK4/6-MEK inhibitor therapy overlapped in wild-type mice, they were statistically different in PC-ko mice with the survival rate for anti-PD-L1 monotherapy now overlapping with vehicle plus IgG controls (Fig. 2D).

Together, these data show that kinase inhibitor therapy concurrently targeting CDK4/6 and MEK is equally effective against *de novo* MPNSTs regardless of plasma cell status. By comparison, plasma cells are essential for an effective MPNST response to anti-PD-L1 ICB therapy, whether as a single agent or when combined with CDK4/6-MEK inhibition.

### Plasma cell-dependent differential gene expression at the single cell level induced by CDK4/6-MEK inhibition in *de novo* MPNSTs

**Fig. 3:**
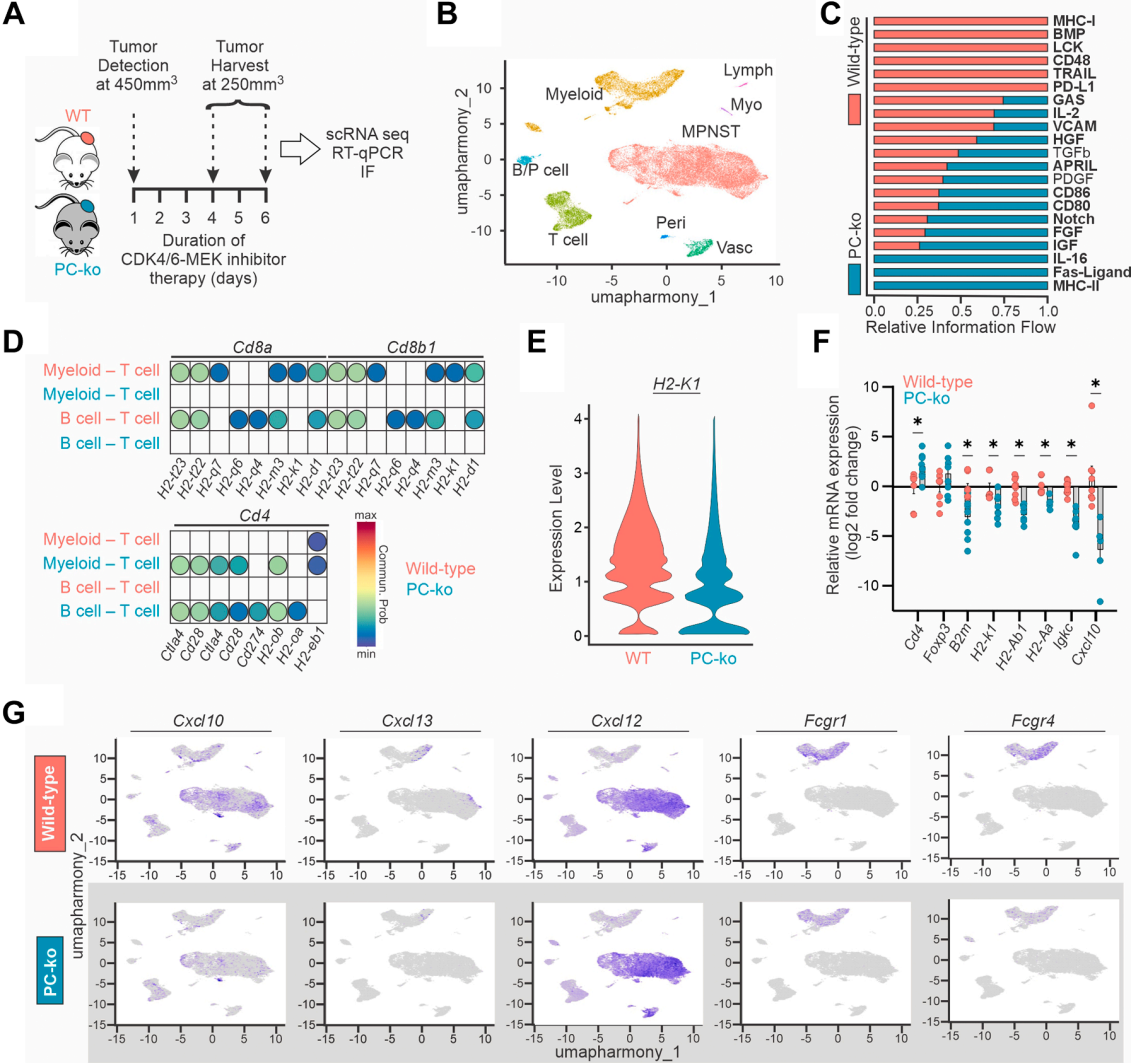
**Loss of plasma cells reduces antigen presentation and predicted engagement of MHC-I by CD8.** Wild-type (WT) and PC-ko MPNSTs, initiated by *Nf1/Ink4a/Arf* editing in the sciatic nerve, were treated daily with CDK4/6-MEK inhibitors (palbociclib at 100 mg/kg, mirdametinib at 1 mg/kg) once tumours reached 450 mm^3^. Tumours were harvested between days 4–6 of therapy when they regressed to 250 mm^3^. (A) Schematic of experimental design. (B) Visualisation of identified cell types from scRNA seq. (C) Top predictions from CellChat for differentially regulated receptor-ligand interactions in WT and PC-ko tumours. (D) Focused bubble plots for CellChat predicted interactions between the indicated ligands and receptors expressed on the Myeloid/B cells and T cells. Commun. Prob., communication probability. (E) Relative expression levels of H2-k1 within the MPNST cluster in WT and PC-ko MPNSTs. (F) Quantitative RT-qPCR validation of selected genes suggested by scRNA seq to be up in WT or PC-ko tumours. Relative mRNA expression was calculated using the delta CT method. (G) Feature plots for visualisation of the expression levels of selected transcripts overlayed onto panel B. Error bars are SEM. P value was obtained via Student’s t test (∗, P < 0.05).

Because MPNSTs in PC-ko mice were not sensitised to ICB therapy by CDK4/6-MEK inhibition, we reasoned that plasma cells must be required to establish a favourable immune environment that primes the tumour for successful immunotherapy. To test that idea, moderately sized *de novo* MPNSTs (∼450 mm^3^) in wild-type and PC-ko mice were treated for 4–6 days with CDK4/6 and MEK inhibitors. This allowed regressing tumours of sufficient size to be harvested for single cell RNA sequencing (scRNA seq) and molecular analyses (Fig. 3A). *FindAllMarkers*^43^ was used to identify cell populations from processed single cell transcriptomes from two wild-type tumours and three PC-ko tumours. The major cell populations identified within the tumours included MPNST cells, myeloid cells, T cells, B/Plasma cells, vascular endothelium, lymphatic endothelium, pericytes, and myocytes (Fig. 3B). Select transcripts used to define each population are listed in Supplementary Fig. S2.

We first focused on plasma cell-dependent gene expression intrinsic to the MPNST population. Non-negative matrix factorisation (NMF) was employed to identify metaprograms (MPs), groups of co-differentially expressed genes (DEGs) (Supplementary Fig. S3A), which represent recurrent programs of intra-tumoural heterogeneity (Supplementary Fig. S3B). Eight MPs were defined, and the top 50 genes in each were extracted for MP identification and downstream analysis. Those metaprograms showing the greatest differences between wild-type and PC-ko MPNSTs were MP1, MP4, and MP5 (Supplementary Fig. S3C). Wild-type tumours containing plasma cells expressed higher levels of transcripts within pathways related to embryonic/neuronal development (MP1) and B/Plasma cell immunity (MP4) whereas PC-ko tumours were enriched for pathways related to myeloid immunity/motility (MP5) (Supplementary Fig. S3D and F).

### Plasma cells promote MHC-I antigen presentation to CD8+ T cells and pro-inflammatory chemokine expression

The two most differentially expressed metaprograms were centered on professional antigen presenting cells, B cells (MP4) and myeloid cells (MP5), suggesting antigen presentation is regulated by plasma cells in MPNSTs (Supplementary Fig. S3B, C, E and F). We used CellChat, a tool to infer and quantify intercellular receptor-ligand interactions from scRNA seq data, to evaluate interactions between major histocompatibility complex class I (MHC-I) or II (MHC-II) antigen presentation molecules and T cell co-receptors, CD8 and CD4.^44^ Strong MHC-I and PD-L1 binding to CD8 and PD-1, respectively, on T cells was exclusive to wild-type tumours (Fig. 3C). The opposite, MHC-II interactions with CD4+ T cells, defined plasma cell-deficient MPNSTs.^45^ Downstream analysis through CellChat infers the strength and cell types involved for each receptor-ligand interaction. MPNSTs in wild-type mice were enriched for interactions between many MHC-I molecules (*H2-t23, H2-k1,* etc.) on myeloid and B cells with *Cd8a/Cd8b1* T cells (Fig. 3D, top). These interactions were absent in PC-ko MPNSTs, which instead presented with more interactions between *Cd4* T cells and MHC-II molecules (*H2-ob, H2-oa, H2-ab1*) (Fig. 3D, bottom).

The predicted receptor-ligand interactions reflected differential expression of the antigen presentation and receptor mRNAs. This included elevated *H2-k1* MHC-I transcripts in wild-type tumours and increased *Cd4* in PC-ko tumours by single cell sequencing (Fig. 3E) and RT-qPCR validation (Fig. 3F), respectively. Unexpectedly, RT-qPCR revealed that PC-ko tumours not only have lower expression of MHC-I (*B2m, H2-k1*) transcripts but also reduced MHC-II molecules (*H2-ab1* and *H2-aa*) (Fig. 3F). This suggests that plasma cells support antigen presentation through both MHC-I and MHC-II molecules, implying that the increase in predicted MHC-II-dependent interactions in PC-ko mice may be a product of increased CD4+ T cells.

Cytokines and chemokines shape the tumour microenvironment by directing immune cell infiltration into tumours. Dimensional reduction and visualisation of the single cell sequencing data revealed elevated expression of *Cxcl10* and *Cxcl1*3 mRNAs in the MPNST and myeloid cells of wild-type tumours versus higher *Cxcl12* in MPNST and vascular cells of PC-ko tumours (Fig. 3G). Both CXCL10 and CXCL13 chemokines contribute to a “hot” tumour microenvironment by recruiting and activating T and B cells, and their expression is required for and positively correlates with better responses to ICB therapy.^46^, ^47^, ^48^, ^49^, ^50^, ^51^, ^52^ By comparison, while CXCL12 can also attract immune cells as part of an inflammatory response, its binding to the CXCR4 receptor activates signalling that promotes tumour growth, angiogenesis, metastasis, and survival.^53^ Consistent with the pro-inflammatory landscape of wild-type tumours, transcripts for *Fcgr1* and *Fcgr4* (low affinity receptors for IgG proteins), were upregulated in myeloid cells of wild-type MPNSTs. Both Fcγ receptors are associated with increased tumour infiltration and activation of immune cells including T cells, and engage with anti-PD-L1 antibodies to augment their *in vivo* activity.^54^^,^^55^

### Plasma cells promote the infiltration and activation of CD8+ T cells within CDK4/6-MEK inhibited MPNSTs

**Fig. 4:**
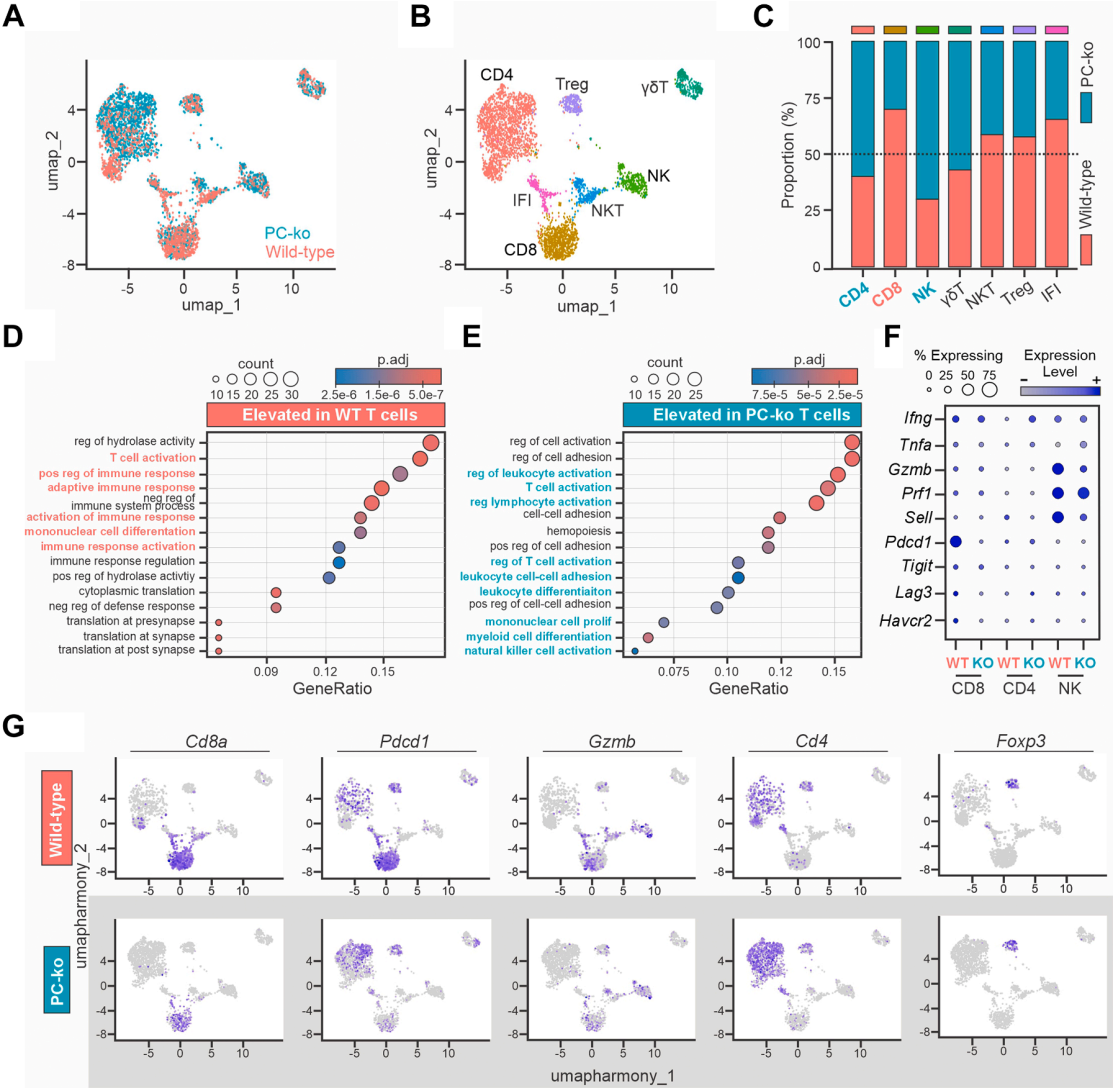
**Plasma cells support the activation of CD8+ T cells.** Analysis of the T cell cluster identified by FindAllMarkers in scRNA seq data from wild-type and PC-ko MPNSTs that were generated and treated as shown in Fig. 3A. (A) Dimensional reduction and visualisation of the T cell cluster split by genotype. (B) FindAllMarkers was used to identify T cell subtypes. (C) Relative amounts of T cell subtypes quantified in WT versus PC-ko mice. Gene Set Enrichment Analysis of biological pathways upregulated in T cells from (D) WT or (E) PC-ko tumours. (F) Relative expression levels of selected T cell exhaustion and activation markers in CD8+ T cells, CD4+ T cells, and NK cells. (G) Feature plots for visualisation of selected transcripts overlayed onto panels A and B.

Immune checkpoint blockades, such as those targeting PD-L1 and PD-1, work by sustaining the activity of anti-tumour T cells.^56^ To better understand how plasma cells promote T cell activation, we isolated the T cell cluster from our scRNA seq dataset for further analysis. Dimensional reduction and visualisation identified multiple transcriptionally distinct subpopulations of T cells with unique patterns of enrichment in wild-type and PC-ko tumours (Fig. 4A). Following clustering on this subpopulation of T cells and identification of DEGs, we identified seven major subpopulations of lymphoid cells which included CD4+ T cells, CD8+ T cells, natural killer (NK) cells, gamma delta T (γδT) cells, NKT cells, regulatory T (Treg) cells, and Interferon-induced T cells (IFI T) (Fig. 4B). Select genes used to delineate subtypes are shown in Supplementary Fig. S4A.

In alignment with CellChat predictions, significantly more *Cd8*-expressing T cells were present in wild-type tumours compared to PC-ko tumours (Fig. 4C). This was mirrored by increases in *Cd4*-expressing T cells as well as NK cells within PC-ko tumours (Fig. 4C). Gene ontology (GO) analysis revealed that T cells from wild-type tumours were enriched for biological pathways related to T cell activation and the adaptive immune response (Fig. 4D). PC-ko tumours also displayed enrichment of T cell activation pathways, as well as gene signatures of activated NK and myeloid cells (Fig. 4E).

CD8+ T cells are known to be activated in response to CDK4/6 and MEK inhibition.^26^ However, an enrichment of T cell activation gene signatures in both wild-type and PC-ko T cells could indicate that plasma cells mediate the activation of specific T cell subsets in response to combined CDK4/6 and MEK inhibition. To assess that possibility, we evaluated the expression of transcripts associated with T cell activation within the T cell cluster. Consistent with cell chat predictions of CD8+ T cell activation, a large fraction of CD8+ T cells from wild-type tumours expressed high levels of *Pdcd1*, which encodes the checkpoint protein, programmed cell death protein 1 (PD-1) (Fig. 4F and G). PD-1 can be a marker of both T cell activation and exhaustion,^57^ therefore we examined the expression of other classical immune checkpoint proteins, Lag3 (*Havcr2*), Tim3, and Tigit. These exhaustion marker mRNAs were expressed at very low levels throughout all T cell subsets, indicating minimal T cell exhaustion in both wild-type and PC-ko tumours at the time of analysis (Fig. 4F). Interestingly, while NK cell numbers were elevated in PC-ko tumours, NK cells in wild-type tumours expressed higher levels of Granzyme B (*Gzmb*) and CD62L (*Sell*). Those changes suggest greater NK cell activation and maturation^58^^,^^59^ in wild-type tumours.

The expression of select T cell transcripts was visualised via feature plots of the T cell population. In agreement with the above findings, increased *Cd8a* expression marked a larger population of CD8+ T cells in wild-type tumours whereas increased *Cd4* defined an enriched CD4+ T cell population in PC-ko tumours (Fig. 4G). *Pdcd1* expression was primarily elevated in CD8+ T cells within wild-type tumours, which also expressed increased *Gzmb* (component of cytotoxic granules that marks activated T [and NK] cells). Some CD4+ T cells expressed *Pdcd1* in PC-ko tumours, but at markedly lower levels than in CD8+ T cells from wild-type tumours. *Foxp3*, a marker of regulatory CD4+ T cells was unchanged between wild-type and PC-ko tumours. These results suggest that plasma cells are essential for the specific recruitment and activation of CD8+ T cells in CDK4/6-MEK inhibitor treated MPNSTs.

### Plasma cells support antigen presentation in myeloid cells within CDK4/6-MEK inhibited MPNSTs

**Fig. 5:**
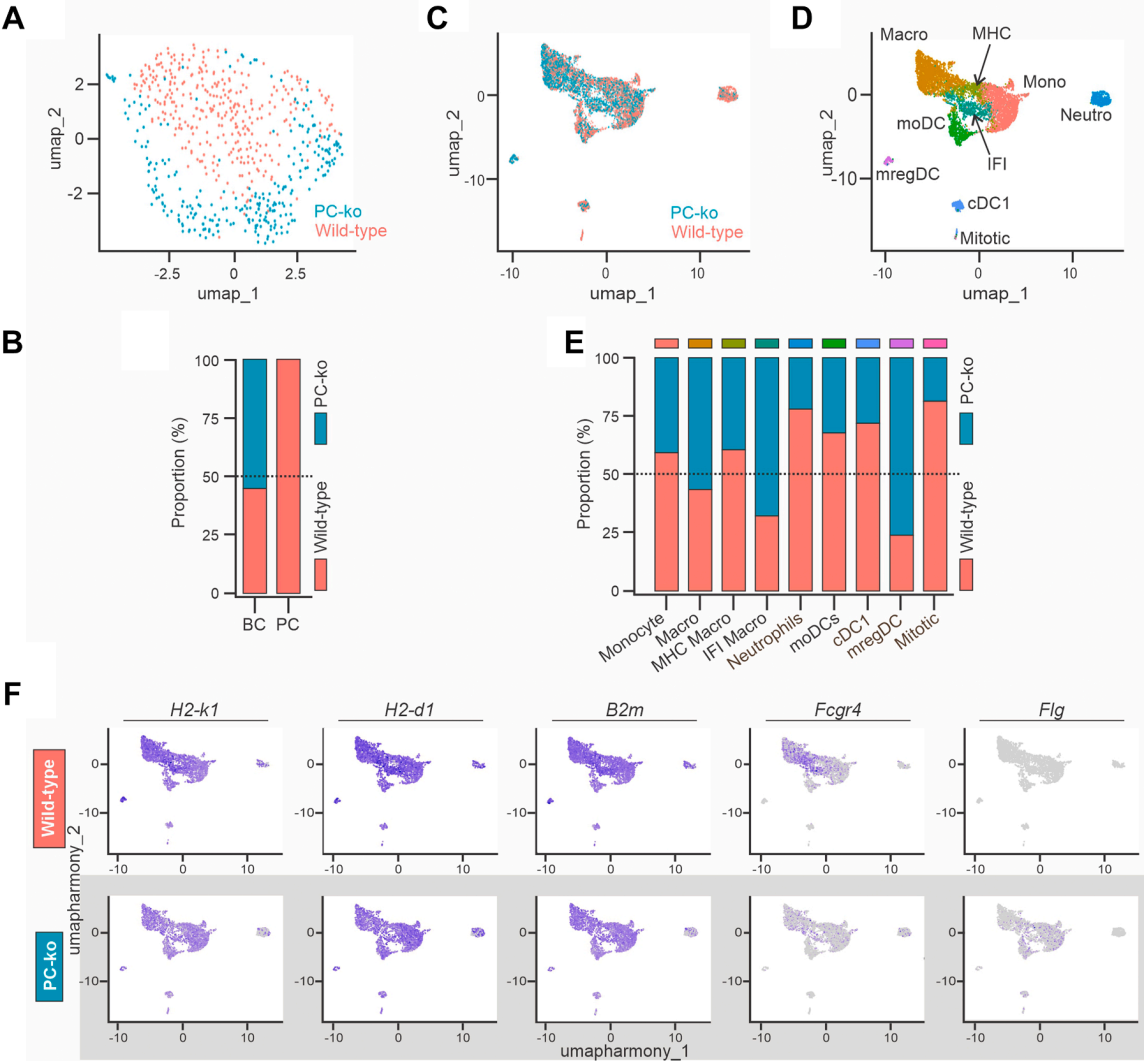
**Plasma cells support antigen presentation and expression of antibody receptors on myeloid cells in CDK4/6-MEK inhibited *de novo* MPNSTs.** B/Plasma cell and myeloid clusters were isolated from scRNA seq data for further analysis. (A) Dimensional reduction and visualisation of B/Plasma cell clusters. (B) Identified subsets are listed and relative amounts in wild-type and PC-ko MPNSTs are quantified. (C) Dimensional reduction and visualisation of the Myeloid cluster. Identified cell types are visualised in (D) with relative amounts of each subtype quantified in wild-type versus PC-ko MPNSTs shown in (E). (F) UMAP Feature plots for visualisation of select transcript expression overlayed onto the myeloid population (see panel C).

The presentation of antigen, either at the tumour cell surface through MHC-I or through antigen presenting cells (APCs) expressing MHC-II, is a critical, early step in T cell activation. We isolated the two main APC clusters from our scRNA seq dataset for characterisation: B/plasma cells and myeloid cells. On the B/plasma cell cluster (Fig. 5A), unbiased clustering delineated B cells from plasma cells and confirmed that plasma cells are only present in wild-type tumours (Fig. 5B). Gene sets used to determine B and myeloid cell subtypes are listed in Supplementary Fig. S4B and C, respectively.

Myeloid cells play important roles in MPNST biology and comprise a large portion of the tumour microenvironment.^60^, ^61^, ^62^, ^63^ The myeloid compartment of MPNSTs is complex and comprised of many cell subpopulations (Fig. 5C and D). Following clustering and DEG identification, nine main subtypes of myeloid cells were defined in our scRNA seq dataset: monocytes, macrophages (Macro), MHC high macrophages (MHC Macro), Interferon-induced macrophages (IFI Macro), Neutrophils, monocyte-derived dendritic cells (moDCs), classical dendritic cells (cDC1), regulatory dendritic cells (mregDC), and mitotic myeloid cells (Fig. 5D).

Many cell types were not affected by plasma cell status, including monocytes, general macrophages, MHC-high macrophages, and interferon-inducible macrophages. Among the altered myeloid populations, neutrophils, moDCs, and cDC1s were significantly reduced in PC-ko tumours (Fig. 5E), suggesting that plasma cells support their presence in tumours. Conversely, PC-ko tumours had enriched mregDCs (Fig. 5E), which famously express immunosuppressive cytokines that promote resistance to ICB therapy.^64^^,^^65^ Visualisation of MHC-I molecules *H2-k1*, *H2-d1*, and *B2m* across all myeloid subsets showed reduced expression of all three transcripts in PC-ko tumours (Fig. 5F). Additionally, myeloid cells from wild-type tumours expressed more *Fcgr4*, which can aid in the uptake of immune complexes by binding the constant region of the antibody. In contrast, myeloid cells in PC-ko MPNST expressed modest levels of *Flg*, which was undetectable in myeloid cells within wild-type tumours. *Flg* encodes filaggrin, a keratin filament binding protein that provides structural support to the skin and maintains its barrier functions.^66^ Of note, recent reports in urothelial carcinoma show that *Flg* expression predicts poor patient response to anti-PD-1 therapy.^67^ Together, these data indicate that plasma cells remodel the myeloid compartment to promote antigen presentation and a pro-inflammatory microenvironment.

### Histological validation of intratumoural immune cell abundance in CDK4/6-MEK inhibited MPNSTs

**Fig. 6:**
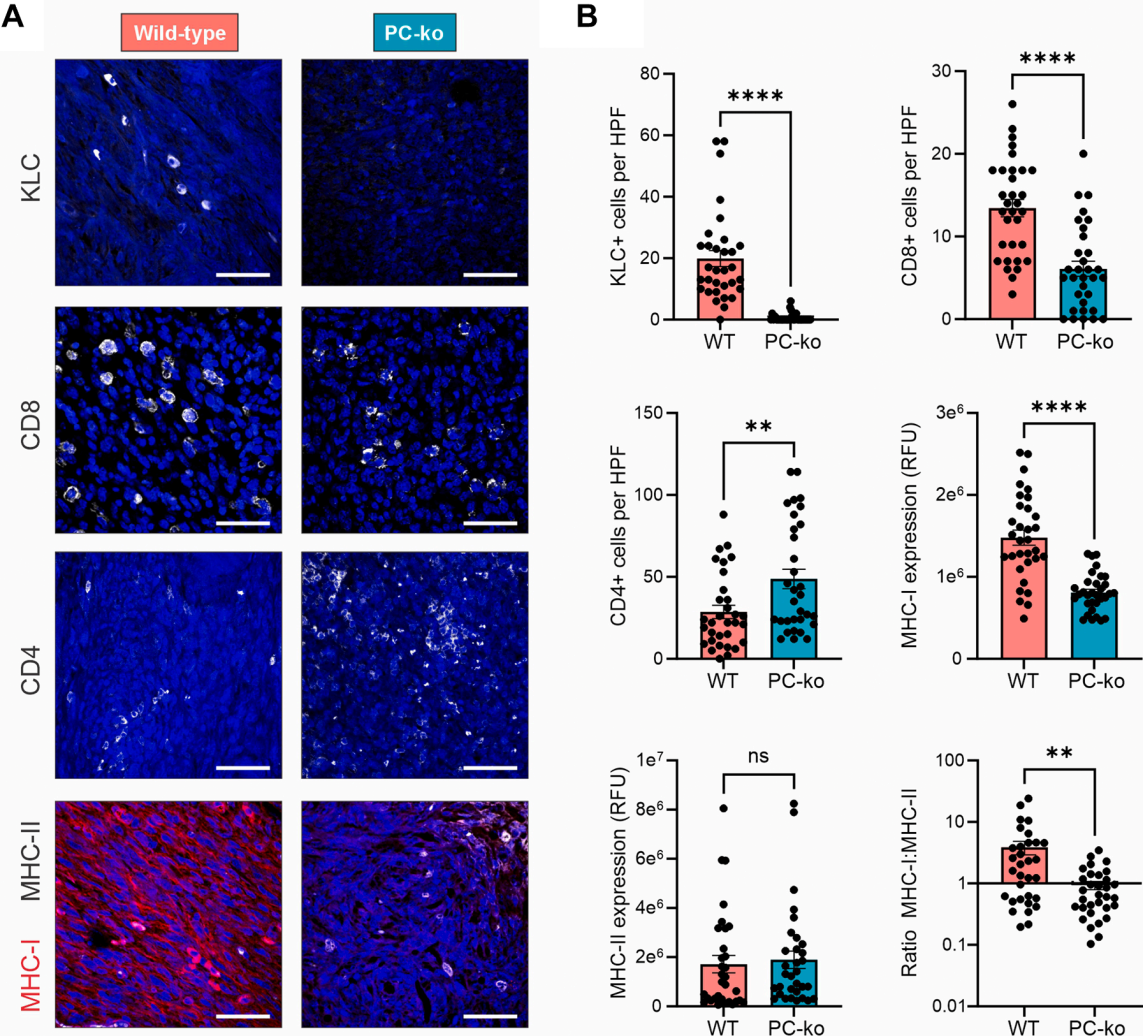
**Plasma cells support MHC-I expression and intratumoural CD8+ T cell populations.** Wild-type and PC-ko MPNSTs were generated and treated as illustrated in Fig. 3a. (A) Representative 40X images showing immunofluorescence (IF) staining of Kappa Light Chain (KLC), CD8, CD4, MHC-I, and MHC-II in *de novo* MPNSTs. White scale bar represents 50 μM. (B) Quantification of IF results from A. Error bars are SEM. P value was obtained via Student’s t test (ns, non-significant; ∗∗, P < 0.01, ∗∗∗∗, P < 0,0001).

Single cell RNA seq findings were first validated at the cellular level and protein levels using immunofluorescent staining of immune cell subsets in the same wild-type and PC-ko MPNSTs that were treated with CDK4/6-MEK inhibitors for 4–6 days. As expected, only wild-type MPNSTs contained kappa-light chain positive (KLC+) cells (Fig. 6A), a specific marker for plasma cells.^68^ In alignment with the scRNA seq data, wild-type MPNSTs had more intratumoural CD8+ T cells (nearly 3-fold) whereas PC-ko tumours had more CD4+ T cells (Fig. 6A). Quantification of positive cells verified statistically significant differences for each cell type in wild-type versus PC-ko tumours (Fig. 6B).

Similarly, as predicted from the single cell sequencing data, significantly higher expression of MHC-I protein was seen in wild-type MPNSTs (Fig. 6A and B). MHC-II protein was not statistically different between the tumour types although it trended to higher levels of expression in PC-ko tumours (Fig. 6A and B). As a result of these differences, wild-type tumours exhibited an increased ratio of MHC-I to MHC-II protein expression relative to PC-ko MPNSTs (Fig. 6B). H&E stained sections of the tumours were analised for potential differences in lymphoid aggregates at the histological level. Immune cell aggregates appeared as diffuse clusters in both wild-type and PC-ko tumours with an overall absence of highly organised regions such as germinal centres or tertiary lymphoid structures (Supplementary Fig. S5A). The total lymphoid aggregate area was quantified and found to be similar in both wild-type and PC-ko MPNSTs (Supplementary Fig. S5A). These findings reveal that plasma cells dictate the composition of the inflammatory immune landscape in CDK4/6-MEK targeted MPNSTs (CD8+ T cells with MHC-I in wild-type versus CD4+ T cells in PC-ko).

Interestingly, wild-type MPNSTs displayed slightly higher PD-L1 expression compared to PC-ko MPNSTs (Supplementary Fig. S5B), which supports the predicted increase in PD-1/PD-L1 signalling in wild-type tumours by CellChat analysis (Fig. 3C). Representative images of negative controls for each immunofluorescent stain are depicted in Supplementary Fig. S5C.

### Plasma cells mediate drug-induced pro-inflammatory immune cell remodeling

**Fig. 7:**
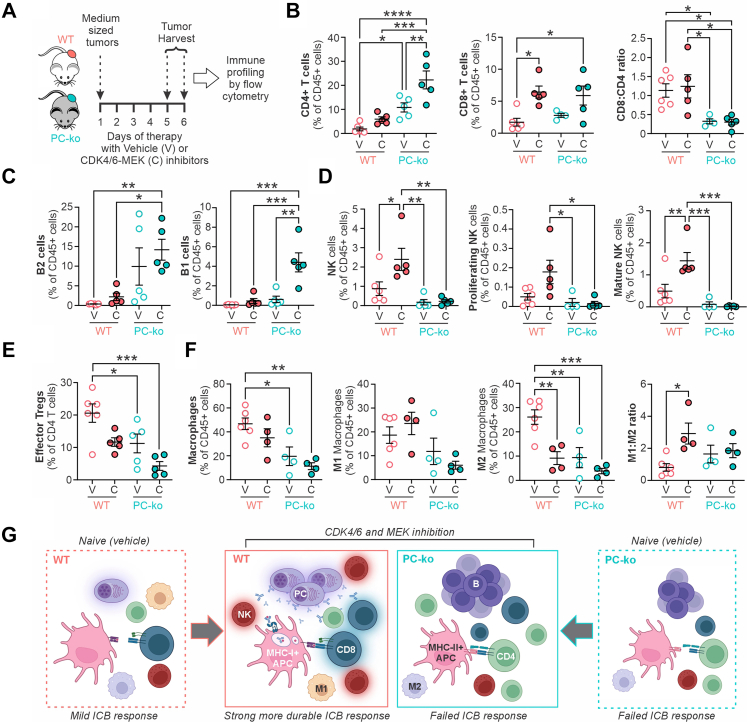
**Plasma cells are required for pro-inflammatory remodeling of the MPNST immune landscape.** (A) Schematic of experimental design. Wild-type and PC-ko MPNSTs were generated as illustrated in Fig. 3A, treated with vehicle or CDK4/6-MEK inhibition for 5–6 days, and intratumoral immune cells quantified by flow cytometric analyses. (B) Left to right: Percent of CD4+ and CD8+ T cells relative to live CD45+ and ratio of CD8+ to CD4+ T cells. (C) Percent of classic B2 cells and innate-like B1 cells relative to live CD45+ cells. (D) Left to right: Percent of total NK cells and highly cytotoxic activated subsets, proliferating and mature, relative to live CD45+ cells. (E) Percent Effector Tregs relative to all CD4+ T cells. (F) Left to right: Percent of total macrophages, M1 macrophages, and M2 macrophages relative to all live CD45+ cells as well as ratio of M1 to M2 macrophages. (G) Model depicting distinct immune environments in plasma cell positive (WT) versus negative (PC-ko) MPNSTs following CDK4/6-MEK inhibition. The schematic illustrates how plasma cells (PCs) increase the recruitment and activation of NK and CD8+ T cells while suppressing M2 macrophages in WT tumours, which corresponds with successful response to ICB therapy. By comparison, PC-ko MPNSTs (which fail to respond to ICB therapy) display a CD4+ T cell, MHC-II antigen presentation, and B cell dominated phenotype. The glow surrounding cells indicates activation. For panels B–F, each dot represents a tumour from an individual mouse. Error bars are SEM. P value was obtained via one-way ANOVA with Tukey’s multiple comparisons. Only statistically significant comparisons are shown (∗, P < 0.05, ∗∗, P, <0.01, ∗∗∗, P < 0.001, ∗∗∗∗, P < 0.0001).

To gain deeper insight into the immune landscapes of wild-type and PC-ko MPNSTs, quantitative flow cytometric immune cell profiling of myeloid and T cell populations was performed in both naïve (vehicle-treated) and CDK4/6-MEK inhibitor treated tumours (Fig. 7A). As in prior experiments, therapy was shortterm for just 5–6 days. One of the more striking observations was that PC-ko tumours exhibited a strong bias toward CD4+ T cell immunity (Fig. 7B), in agreement with our earlier results from single cell RNAseq and histological analyses. This was evident even at baseline with PC-ko tumours displaying higher CD4+ T cell infiltrates compared to wild-type tumours. In addition, CDK4/6-MEK inhibition selectively increased CD4+ T cells only in knockout MPNSTs. Wild-type MPNSTs, by comparison, exhibited a pronounced CD8+ T cell infiltration bias. Interestingly, while kinase inhibition significantly increased CD8+ T cell infiltration within wild-type tumours, the ratio of CD8:CD4 T cells was unchanged by drug treatment in both wild-type and PC-ko MPNSTs. This reveals that the T cell subtype biases are inherently skewed toward CD8+ in wild-type tumours versus CD4+ in PC-ko tumours.

Several other noteworthy differences in immune cell infiltrates between wild-type and PC-ko MPNSTs were identified. Intratumoral B cell accumulation was negligible in wild-type tumours, both naïve and drug-treated, whereas it was markedly induced by therapy in PC-ko MPNSTs (Fig. 7C). This was apparent for both conventional B2 cells that are part of the adaptive immune response as well as innate-like B1 cells. In contrast, NK cell infiltration was only induced by CDK4/6-MEK inhibitor therapy in wild-type MPNSTs while NK cells remained low to undetectable in PC-ko tumours (Fig. 7D, left panel). The drug-induced rise in cytotoxic proliferating (Fig. 7D, middle panel) and mature (Fig. 7D, right panel) NK cells reflects their activation in wild-type tumours, consistent with scRNAseq data (Fig. 4F). No changes in the precursor (uncommitted progenitors with limited cytolytic activity) and terminal (fully mature and highly cytotoxic) NK cell populations were seen in either wild-type or PC-ko tumours (Supplementary Fig. S6), although terminal NKs trended upward in therapy treated wild-type tumours. Together with the T cell analyses, these data show that the drug-induced increases in CD8+ T cells and NK cells in wild-type MPNSTs are plasma cell-dependent events.

Immunosuppressive cell types were also assessed. Effector regulatory T cells (CD4+ Tregs) were noticeably more prevalent in untreated wild-type MPNSTs relative to PC-ko MPNSTs, both in the absence or presence of CDK4/6-MEK inhibitor therapy (Fig. 7E). MPNSTs are macrophage-rich tumours,^63^ as seen in wild-type untreated tumours (Fig. 7F, left panel), but the absence of plasma cells in PC-ko tumours correlated with a significant reduction in total macrophages. Unexpectedly, CDK4/6-MEK inhibition had no effect on the accumulation of pro-inflammatory M1 macrophages in both wild-type and PC-ko tumours; however, treatment selectively reduced the levels of immune suppressive M2 macrophages in wild-type tumours (Fig. 7F, middle panels). As a result, CDK4/6-MEK inhibition promoted a pronounced increase in the M1:M2 ratio only within wild-type MPNSTs (Fig. 7F, right panel), reflecting a shift toward an anti-tumour immune response in those tumours. These findings strongly suggest that the failure of PC-ko tumours to respond to ICB therapy cannot be explained by an enhanced immunosuppressive environment since immune suppressive cell types (both Tregs and M2 macrophages) are poorly represented within those tumours.

As modelled in Fig. 7G, our collective data indicate that plasma cells remodel both adaptive and innate immune cells to promote a pro-inflammatory state in MPNSTs and enable successful ICB therapy. This remodeling, which was induced by CDK4/6-MEK inhibitor therapy, is characterised by increased infiltration and activation of NK and CD8+ T cells, MHC-I antigen presentation, and a heightened M1 macrophage presence relative to M2 macrophages. By comparison, tumours lacking plasma cells are unresponsive to ICB therapy and have an immune phenotype dominated by B and CD4+ T cells with MHC-II antigen presentation. Overall, a failure to engage pro-inflammatory and cytotoxic immune machinery, as opposed to enhanced immunosuppression, embodies the PC-ko phenotype.

### B and plasma cell abundance prognosticates enhanced survival in human sarcomas

To assess the importance of plasma cells in patient outcome, we queried The Cancer Genome Atlas Sarcoma (TCGA SARC) bulk RNA sequencing (RNA-seq) dataset and predicted relative immune cell subsets using the CIBERSORT algorithm.^69^ The cell fraction for each subtype was identified for all sarcomas while samples without a detectable fraction of plasma cells were excluded from downstream analysis. In many other cancers, plasma cells co-organise with immune cells that promote anti-tumour immunity including B cells, CD4+ helper T cells, and CD8+ cytotoxic T cells.^28^^,^^29^ In agreement with this, the plasma cell fraction in sarcomas positively correlated with each of those cells while being inversely correlated with pro-tumourigenic cell types, such as M2 macrophages and mast cells (Supplementary Fig. S7A).

Intratumoural plasma cells are associated with prolonged survival in most cancers,^27^, ^28^, ^29^, ^30^ prompting us to perform a Kaplan–Meier survival analysis of the TCGA SARC dataset. Samples were binned into the upper and lower 45th percentiles based off their relative plasma cell fraction. Patient survival was significantly extended in patients with a higher intratumoural plasma cell signature (Supplementary Fig. S7B). Surprisingly, a higher fraction of CD8+ T cells did not correlate with longer patient survival (Supplementary Fig. S7C). Because CD8+ T cells and plasma cells are proportional with each other in these tumours (Supplementary Fig. S7A), we then compared the survival of patients with high versus low fractions of both cell types. Patients with elevated intratumoural CD8+ T cell and plasma cell signatures trended to having longer survival times than those with lower fractions, although this difference did not reach statistical significance (Supplementary Fig. S7D). It should be noted that a limitation of this dataset is its broad representation of all sarcoma types since patients with MPNSTs only constitute a minor fraction of the total cohort. Nonetheless, these findings suggest that increased intratumoural plasma cells are more valuable prognostic markers than CD8+ T cells for prolonged patient survival in sarcomas, a conclusion that has been drawn for other tumour types.^28^, ^29^, ^30^

## Discussion

MPNSTs have an immune suppressive tumour microenvironment and do not respond well to any therapy, including ICB immunotherapy. We recently reported that MPNSTs can be sensitised to anti-PD-L1 therapy by CDK4/6-MEK inhibition, which uniquely stimulates plasma cell accumulation in the tumours coincident with CD8+ T cell activation. Combined targeting of CDK4/6, MEK, and PD-L1 provides durable, “curative” responses in a small portion of mice, which has never been achieved by any therapeutic in this preclinical model. Here, we directly tested the importance of plasma cells to the ICB response in MPNSTs and found they are vital for successful immunotherapy.

Our findings demonstrate a causal relationship between plasma cells and the tumour immune response to ICB therapy. In many cancers, B and plasma cells are increasingly recognised as robust prognosticators of prolonged patient survival^27^, ^28^, ^29^, ^30^ and improved response to ICB agents.^28^^,^^31^, ^32^, ^33^, ^34^ However, the role of plasma cells in therapy-induced anti-tumour immunity has never been investigated and only a few studies have explored the contributions of B cells. Genetic ablation or antibody depletion of all B-lineage cells greatly reduced the anti-tumour effects of ICB monotherapy as well as ICB combination therapies in preclinical tumour models.^21^^,^^40^ Griss et al. evaluated patients with melanoma and likewise found that B cells predicted response to ICB therapy and were required for establishing a pro-inflammatory state in the tumours.^41^ They found that newly differentiated B cells, called plasmablasts (which are lost in B cell deficient mice), were crucial for producing cytokines for T cell recruitment. Plasmablasts not only secrete cytokines, they also make antibodies (albeit at lower levels than mature plasma cells) and internalise antigens for presentation to T cells (a function lost by mature plasma cells).^70^^,^^71^ Because plasmablasts are still present in *AID−/−; μS−/−* mice, which only lack mature plasma cells, our data suggest it is the mature plasma cells that mediate the anti-tumour response to anti-PD-L1 therapy in MPNSTs. Notably, the mice employed in this study were *Nf1*^+/+^ and as such the *de novo* MPNST model mimics sporadic MPNST development, which accounts for 50% of all MPNSTs. The other 50% of MPNSTs arise in people with Neurofibromatosis Type 1 (NF1) in which germline *NF1* gene mutation is inherited in one allele. It is currently unknown how *NF1* haploinsufficiency within plasma cells may alter their ability to sensitise MPNSTs to ICB therapies.

Plasma cells are best known for producing antibodies, which carry out many potential roles in the tumour microenvironment (TME) that are incompletely understood.^72^ First, antibodies can activate the complement cascade, which causes immunologic, osmotic death through the formation of pores in the cell membrane.^73^ Second, antibodies can promote lysis of target cells by triggering natural killer (NK) cell activation. That process, termed antibody-dependent cellular cytotoxicity (ADCC), relies on the binding of antibody Fc regions to Fcγ receptors on NK cells.^74^ Indeed, *Fcgr4* was upregulated in wild-type MPNSTs and encodes FcgRIV, an essential mediator of ADCC.^75^ This is consistent with a critical role for NK cells in mediating the plasma cell-dependent response to ICB therapy given the significant increase in cytotoxic NK cell infiltration that was induced by CDK4/6-MEK inhibition within wild-type, but not PC-ko, MPNSTs. Finally, antibodies can label tumour cells for phagocytosis or form immune complexes to aid antigen presentation by phagocytes.^73^ Many of these pathways were selectively upregulated in wild-type, CDK4/6-MEK targeted MPNSTs according to NMF analysis, but not in PC-ko tumours. Thus, intratumoural plasma cells may support CD8+ T cell activation by forming immune complexes with antigen for uptake and presentation by APCs.

While typified by their mass production of antibodies, plasma cells may promote sensitivity to ICB therapy in an antibody-independent manner. In that regard, transcripts expressing chemokines (*Cxcl10, Cxcl13*) and antigen presentation molecules (*H2-k1, B2m, H2-Aa*) were notably upregulated in wild-type MPNST and myeloid cells treated with CDK4/6 and MEK inhibitors relative to tumours lacking plasma cells. Those intratumoural changes are widely known to support infiltration and activation of immune cells (B, T, myeloid, and dendritic cells) which may, over time, assemble with plasma cells into mature tertiary lymphoid structures (TLS) that heighten CD8+ T cell activity. Intriguingly, it has been demonstrated that human macrophages stimulate plasma cell differentiation through Cxcl10 expression and intercellular contact, partially mediated by Vascular Cell Adhesion Molecule 1 (VCAM-1).^76^ Thus, Cxcl10 may play a multifaceted role in the MPNST response to ICB therapy by recruiting CD8+ T cells and sustaining intratumoural plasma cells. This is supported by plasma cell-deficient tumours harbouring fewer CD8+ T cells and little *Cxcl10* expression.

Histological analyses of the CDK4/6-MEK targeted MPNSTs revealed loosely clustered lymphoid aggregates of similar morphology and size in both wild-type and plasma cell-deficient mice. We observed no evidence of bona fide TLS formation. We speculate the diffuse lymphoid aggregates detected are pre-TLS in nature, suggesting that 4–6 days of kinase inhibitor therapy is an insufficient length of time to induce TLS formation. It will be interesting to determine in future studies if the number and/or location of intratumoural plasma cells within kinase inhibitor-induced TLSs (emerging versus mature) dictates the magnitude and duration of successful ICB therapy.

As MPNSTs develop, they become increasingly “cold” immunologically with reduced antigen presentation, exclusion and/or exhaustion of infiltrating T cells, and preponderance of pro-tumourigenic M2 macrophages, which promotes tumour resistance to ICB therapies.^77^ We found that dual CDK4/6-MEK inhibition for just 4–6 days in wild-type MPNSTs was sufficient to activate a pro-inflammatory, MHC-I directed CD8+ T cell and NK cell response. That rapid remodeling of the immune milieu favourably corresponded with sustained anti-tumour effects of anti-PD-L1 therapy and significantly improved survival. Conversely, MPNSTs lacking plasma cells were strongly biased toward a CD4+ T cell immune cell phenotype with elevated B cells and MHC-II antigen presentation. CD4+ T cell recruitment may be a result of tumour-resident myeloid or B cells attempting to compensate for the lack of plasma cells; however, these tumours still fail to respond to ICB therapy. It is currently unknown how CDK4/6 and MEK inhibitors augment normal plasma cell biology directly yet our studies demonstrate that ICB sensitivity prompted by dual CDK4/6-MEK inhibition is dependent on plasma cells. An enriched CD8+ T cell response and bolstered antigen presentation have been noted in other tumour types sensitised to immune checkpoint blockades, either through chemotherapy and MEK inhibition^78^ or epigenetic therapies.^79^

Others have also shown that combined inhibition of CDK4/6 and MEK triggers a strong anti-tumour response in Ras-driven pancreatic adenocarcinoma that depended on CD8+ T cell activation.^26^ Plasma cells were not examined in the above models, but our work strongly suggests that they likely mediated or contributed to the successful remodeling of the immune landscape and sensitisation to ICB therapies in those settings. Here, we show that plasma cells are required for pro-inflammatory changes in adaptive and innate immunity in response to CDK4/6-MEK inhibition in MPNSTs. Moreover, plasma cells are needed to sensitise *de novo* MPNSTs to ICB therapy and they predict a modest survival benefit in independent sarcoma cohorts. Both in preclinical studies and patient tumours, higher plasma cell fractions predict favourable clinical outcomes, highlighting the translational relevance of this MPNST model. Future studies examining the individual or combined contributions of the plasma cell-dependent, drug-induced immunological changes should be highly informative and may guide strategies to combat acquired resistance which sadly occurs at an undesirably high rate.

Our analyses of tumour infiltrating immune cells paired with CellChat intercellular receptor-ligand interaction predictions revealed that CD8+ T cell infiltration and activation in CDK4/6-MEK targeted MPNSTs is a plasma cell-dependent process. Two of the strongest receptor-ligand interactions observed exclusively in wild-type tumours were for CD8+ T cell receptor associations with MHC-I and lymphocyte-specific protein tyrosine kinase (LCK). LCK plays a central role in T cell activation and anti-tumour immunity. It is critical for remodeling the TME in melanoma and predicts response to immunotherapy.^80^ Across multiple cancers, LCK levels positively correlate with CD8+ T cell tumour infiltration.^81^ Tumour necrosis factor-related apoptosis-inducing ligand (TRAIL), which had strong predicted interaction with death receptors in wild-type MPNSTs, is upregulated on activated T cells and contributes to T cell killing of tumour cells.^82^ Conversely, dominant receptor-ligand interactions in PC-ko MPNSTs included MHC-II with CD4, IL-16 (a chemoattractant for CD4+ cells), and Fas ligand (FasL), which is highly expressed in some MPNSTs and has a complex role in anti-tumour immunity. Endothelial FasL is linked with an absence of CD8+ T cells due to preferential killing of those cells.^83^ Defining the essential mechanisms by which plasma cells augment the T cell response may be crucial for delineating which MPNSTs or other solid tumours are likely to respond well to ICB therapy.

In addition to the immunological effects of plasma cells in MPNST therapy response, we saw increased embryonic and neuronal development gene expression in wild-type tumours. This could reflect an unwanted increase in stem-like tumour cells within the drug-treated MPNSTs. By activating cytotoxic NK and CD8+ T cells that can effectively clear a large portion of the tumour cells, CDK4/6-MEK inhibition could cause an undesirable enrichment for a stem-population less affected by cell cycle-targeting therapies, potentially driving drug resistance. Other transcripts associated with cancer stem cells, like *Fos*^84^ and *Dusp1*,^85^ were concurrently elevated. Our data also predict an upregulation of hepatocyte growth factor (HGF) signalling in wild-type tumours. HGF signals through its receptor, MET, a tyrosine kinase receptor which is a marker of stemness in glioblastoma,^86^ is genetically amplified along with *HGF* in NF1-related MPNSTs^87^ and is sufficient for Schwann cell dedifferentiation into MPNSTs.^14^ These observations are relevant because most tumours in wild-type mice (85–90%) treated with CDK4/6, MEK, and PD-L1 targeted therapy ultimately develop resistance and regrow even though 10–15% of mice are cured (^20^ and data herein). It is possible that therapy-resistant stem-like MPNST cells residing in the TME are enriched upon immune cell destruction of other tumour cells and then reinvigorate the MPNST population. Further analysis of therapy sensitive versus resistant MPNSTs should provide valuable insights into the role of stem-like MPNST cells in resistance to therapies targeting CDK4/6, MEK, and PD-L1. Therapeutic modification of the tumour microenvironment may be better optimised through drug sequencing and result in a more profound response to ICBs in MPNST as it does in preclinical models of colorectal cancer.^88^

In summary, we show that plasma cells are required for *de novo* MPNSTs to respond to anti-PD-L1 therapy and they predict favourable outcomes in both preclinical and clinical settings. Tumour-resident plasma cells function, at least in part, by remodeling the MPNST immune landscape into a pro-inflammatory NK and CD8+ T cell milieu with increased MHC-I antigen presentation and reduced immunosuppressive M2 macrophages. That immune state is primed for an efficacious response to ICB agents. Our findings highlight two key points. First, tumour resident plasma cells may be pivotal in predicting which patients will respond to ICB therapies. Second, anticancer treatments that increase intratumoural plasma cells may be a key step in converting cold tumours into “hot” tumours that will respond effectively to ICB therapy. These points have increasing clinical relevance that likely extends beyond MPNSTs to other solid tumours because trials involving ICB therapies, particularly combinations with chemotherapeutic or targeted agents, are on the rise.

### Limitations of the study

While intratumoural plasma cells are shown to promote a pro-inflammatory tumour environment in response to CDK4/6-MEK inhibition, the precise mechanisms underlying the immune remodeling remain to be defined. Further study is also warranted to determine the relative contributions of the plasma cell-dependent changes in cytokines and immune cell populations to an effective ICB response.

How well our findings can be generalised to other solid tumours and anticancer treatments is currently not known. Comprehensive analyses of the importance and function of plasma cells in mediating successful ICB therapy for other cancer types will be informative. Likewise, drug screens should be valuable in identifying additional cancer therapeutics (either used alone or in combination) that are capable of inducing intratumoural plasma cells.

## Contributors

JJL and DEQ conceived and designed the studies. JJL, AK, ZRZ, ELH, CA, JAR, CAK, ECE, and EV conducted experiments and acquired data. JJL, RR, AK, ZRZ, ELH, JAR, EV, DKM, PB, RDD, JCH, BWD, and DEQ analysed the data. JJL and DEQ accessed and verified the underlying data. RR, AJ, CRW, AWB, and NJK provided key reagents. JJL, RDD, BWD, and DEQ obtained funding. JJL, RR, DKM, RDD, JCH, BWD, and DEQ provided observations and scientific interpretations. All authors reviewed and approved the manuscript.

## Data sharing statement

Mouse tumour single cell RNA seq data will become publicly available in the Gene Expression Omnibus (GSE306038) upon publication. All other data generated in this study are available upon request from the corresponding author.

## Declaration of interests

The authors declare no competing interests.
