# Supplementary material for: Intratumoural plasma cells mediate a pro-inflammatory response to kinase inhibition and successful immune checkpoint blockade therapy in malignant peripheral nerve sheath tumours
Source: eBioMedicine. 2026 Aug 1;131:106413. doi: 10.1016/j.ebiom.2026.106413 (PMC13453594; doi:10.1016/j.ebiom.2026.106413)
Supplement: Supplementary file 1 [file mmc1.pdf]

Supplementary Data

Joshua J. Lingo et al.,

"Intratumoural plasma cells mediate a pro-inflammatory response to kinase inhibition and successful immune checkpoint blockade therapy in malignant peripheral nerve sheath tumours."

Supplementary Fig. S1

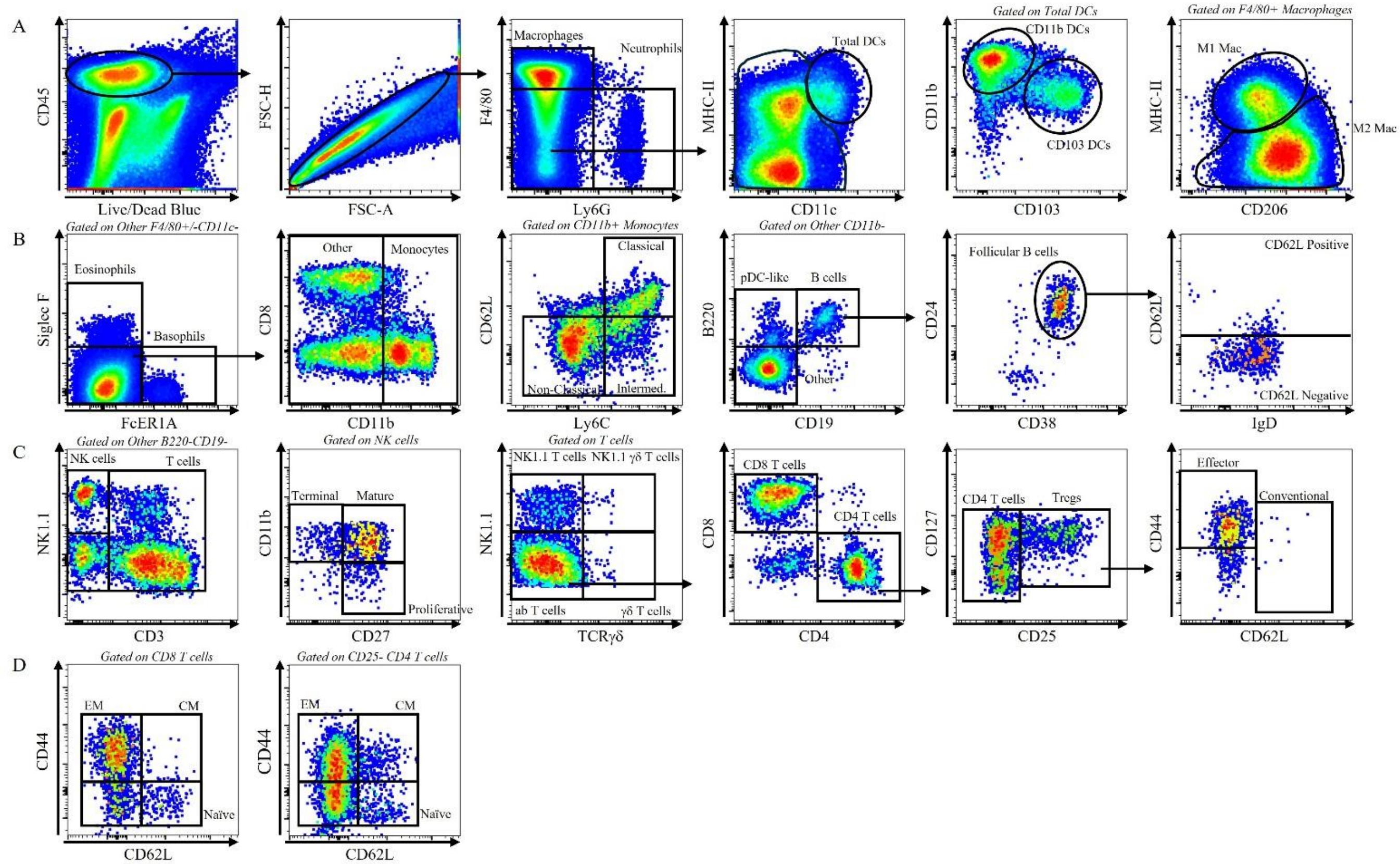

Supplementary Fig. S1: Gating Strategy for flow cytometric profiling of intratumoural immune cells. (A) After gating on live $CD45^+$ cells and excluding doublets, macrophages were identified by high F4/80 expression, and neutrophils by Ly6G expression. Macrophages were further characterized based on MHC-II and CD206 expression to define M1- and M2-like polarization states. Dendritic cells (DCs) were identified from the $F4/80^-Ly6G^-$ population by MHC-II and CD11c expression, and further subdivided into $CD11b^+$ and $CD103^+$ DCs. (B) Within the $F4/80^{+/-}$ $CD11c^-$ population, eosinophils were identified by Siglec-F expression and basophils by FcεR1α expression. From the Siglec-F^-FcεR1$α^-$ population, monocytes were defined by high CD11b expression and further classified into classical, intermediate, and non-classical subsets based on CD62L and Ly6C expression. From the CD11b^neg/lo population, pDC-like cells and B cells were identified using B220 and CD19 expression. B cells were further subdivided into $CD62L^+$ and $CD62L^-$ follicular populations using CD24, CD38, CD62L, and IgD. (C) Non-B cells were classified into NK cells and T cells based on NK1.1 and CD3 expression. NK cell maturation/activation states (terminal, mature, and proliferative) were defined by CD11b and CD27 expression. $NK1.1^+$ T cells, $NK1.1^+$ γδ T cells, and γδ T cells were distinguished from αβ T cells based on NK1.1 and γδ TCR expression. Within the αβ T cell compartment, $CD4^+$ and $CD8^+$ T cells were identified, and CD25 expression was used to define $CD4^+$ regulatory T cells (Tregs). Treg activation status was assessed using CD44 and CD62L expression. (D) Finally, $CD8^+$ T cells and $CD25^-$ $CD4^+$ T cells were further subdivided into memory subsets based on CD44 and CD62L expression.

Supplementary Fig. S2

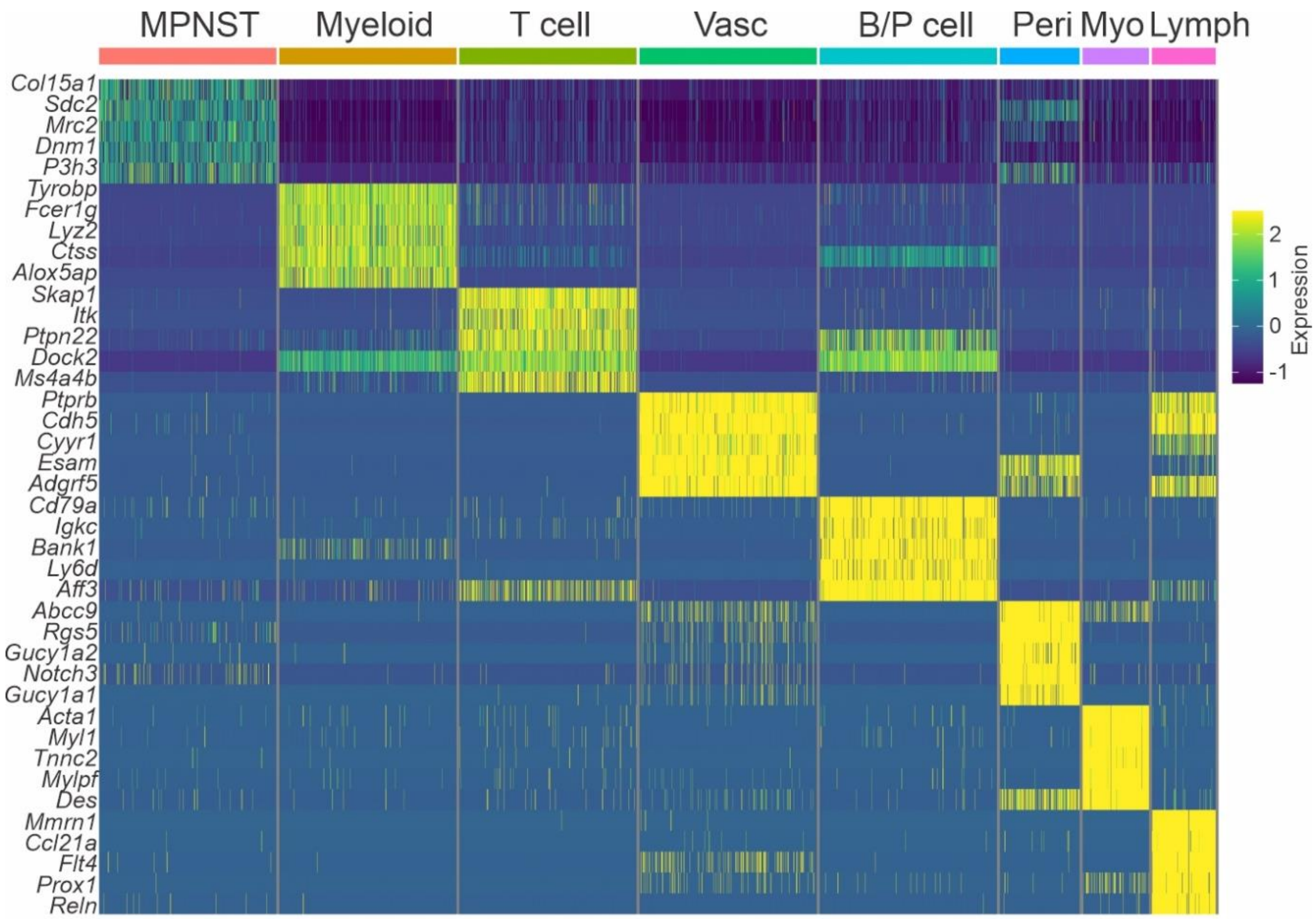

Supplementary Fig. S2. Identification and delineation of major cell populations in *de novo* MPNSTs. Heatmap showing gene expression subsets, obtained from FindAllMarkers analysis of scRNA seq data, which were used to define the different major cell populations in *de novo* MPNSTs from WT and PC-ko mice that had been treated for 4 to 6 days with CDK4/6 and MEK inhibitors.

Supplementary Fig. S3

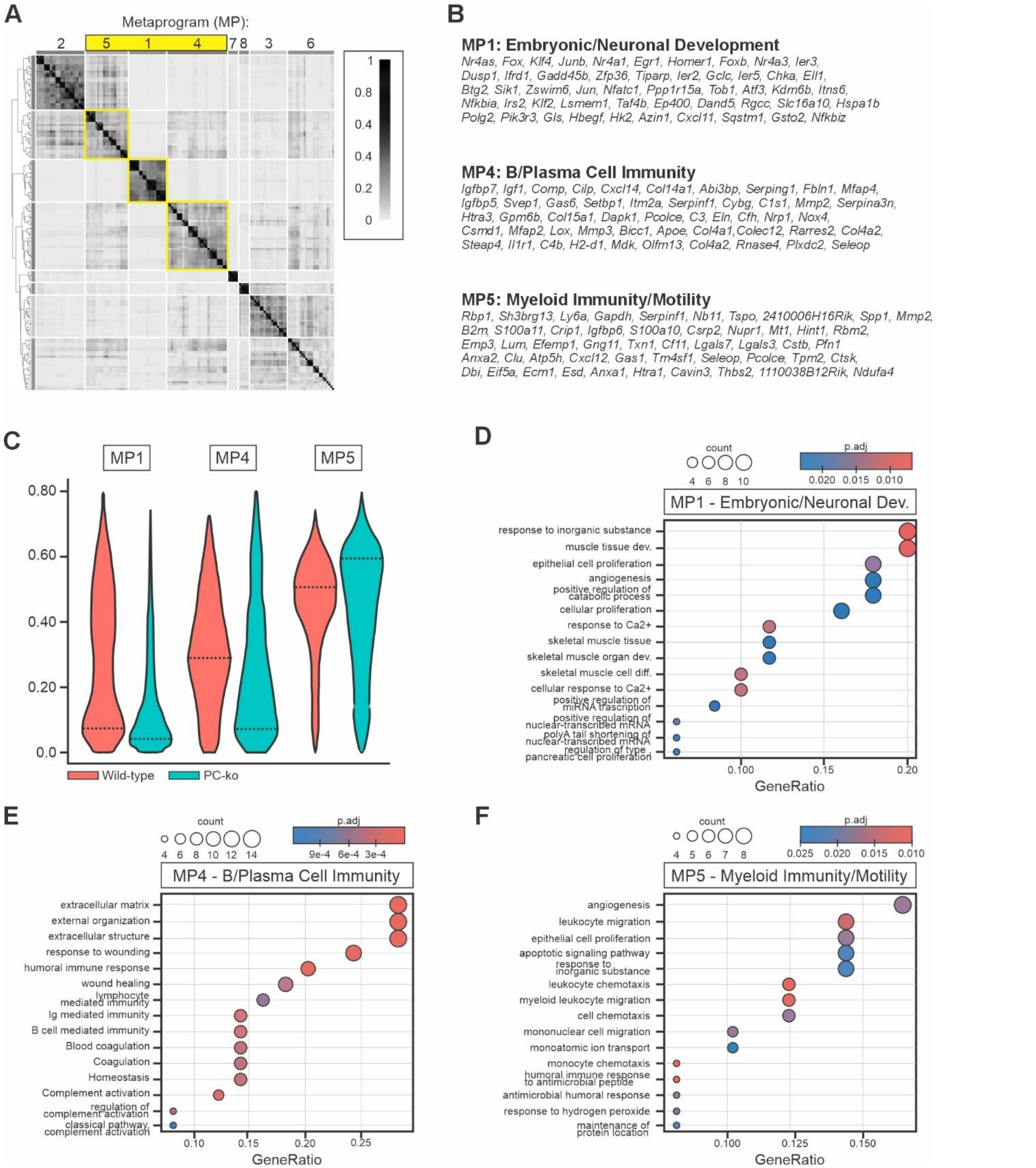

Supplementary Fig. S3: Non-negative matrix factorization of MPNST population clusters. Non-negative matrix factorization (NMF) of scRNA seq data was employed to identify co-occurring panels of transcripts in WT and PC-ko MPNSTs that had been treated for 4-6 days with CDK4/6-MEK inhibitors. (A) Greyscale heatmap of identified metaprograms (MPs) by NMF analysis with yellow highlighting those (MP1, MP4, and MP5) with significant differences between WT and PC-ko tumors. (B) List of the top 50 genes from MPs 1, 4, and 5. (C) Relative expression levels of MP1, MP4, and MP5. Panels D-F: Dot plots showing enriched pathways based off the expression of the transcripts for

MPs 1, 4 and 5, as determined by gene set enrichment analysis (GSEA). (D) MP1 – Embryonic/Neuronal Dev (development). (E) MP4 – B/Plasma Cell Immunity. (F) MP5 – Myeloid Immunity/Motility.

Supplementary Fig. S4

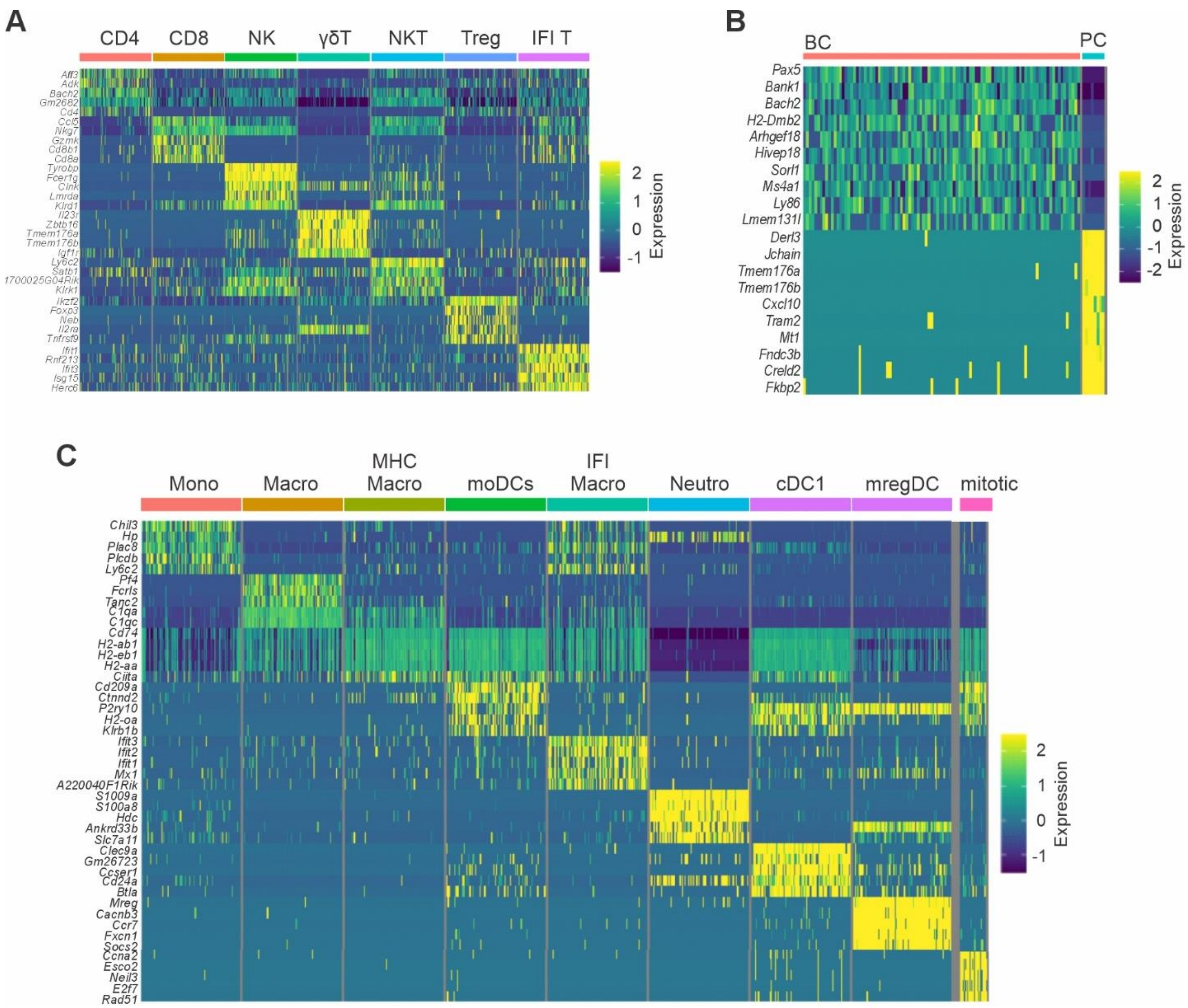

Supplementary Fig. S4: Delineation of immune cell subsets for T cell, B/Plasma cell, and myeloid clusters in CDK4/6-MEK inhibited *de novo* MPNSTs. FindAllMarkers analysis of scRNA seq data was used to determine immune cell subsets within immune cell clusters in WT and PC-ko MPNSTs that had been treated for 4-6 days with CDK4/6-MEK inhibitors. Heatmaps of relative gene expression are listed for the: (A) T cell cluster (Natural killer cells, NK; gamma-delta T cell, γδT; NKT cell, NKT; Regulatory T cell, Treg; and Interferon induced T cells, IFI T), (B) B/Plasma cell cluster (B cell, BC; Plasma cell, PC), and (C) the myeloid cluster (Monocyte, Mono; Macrophage, Macro; MHC-hi Macrophage, MHC Macro; monocytic dendritic cells, moDCs; Interferon induced macrophages, IFI Macro; Neutrophils, Neutro; classical DCs, cDC1; regulatory DCs, mregDC; proliferating myeloid cells, Mitotic).

Supplementary Fig. S5

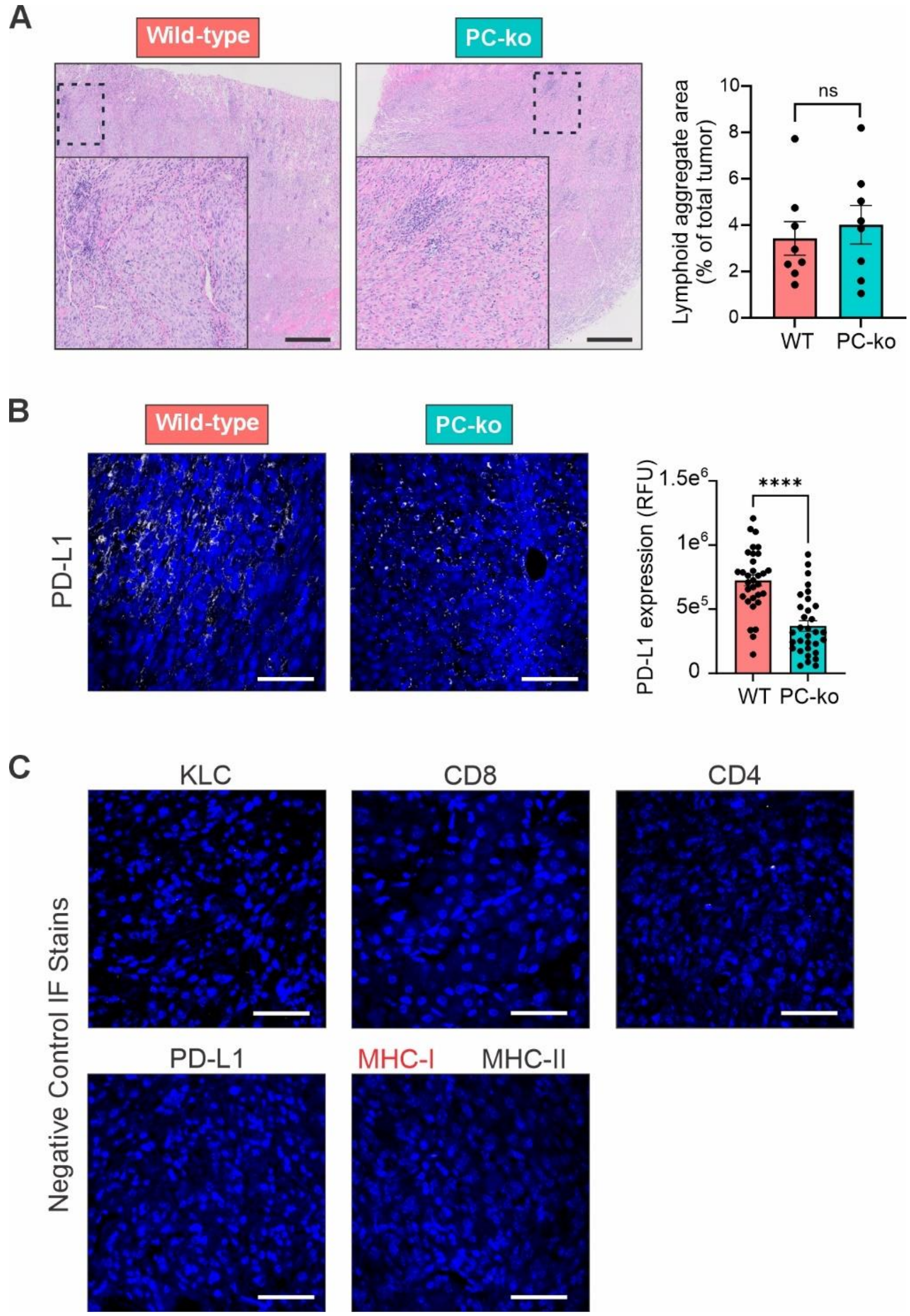

Supplementary Fig. S5: Additional histopathological analyses of CDK4/6-MEK inhibited *de novo* MPNSTs. (A) Representative 10X images of H&E stained wild-type (WT) and PC-ko tumors with quantified area for lymphoid aggregates graphed to the right. Magnified region corresponds to the area in the black dashed box. Black scale bar represents 500 µM. Error bars are SEM. Statistical comparison done by Student's t test; ns, not significant. (B) Representative 40X images of IF staining for PD-L1 in WT and PC-ko *de novo* MPNSTs with quantified data shown to the right. White scale bar represents 50 µM. Error bars are SEM. P value was obtained via Student's t test (****, $P < 0.0001$). (C) Representative 40X images of IF staining results for negative control stains (no primary antibody) for each immunostaining protocol that employed distinct secondary antibodies. The target for each stain is indicated.

Supplementary Fig. S6

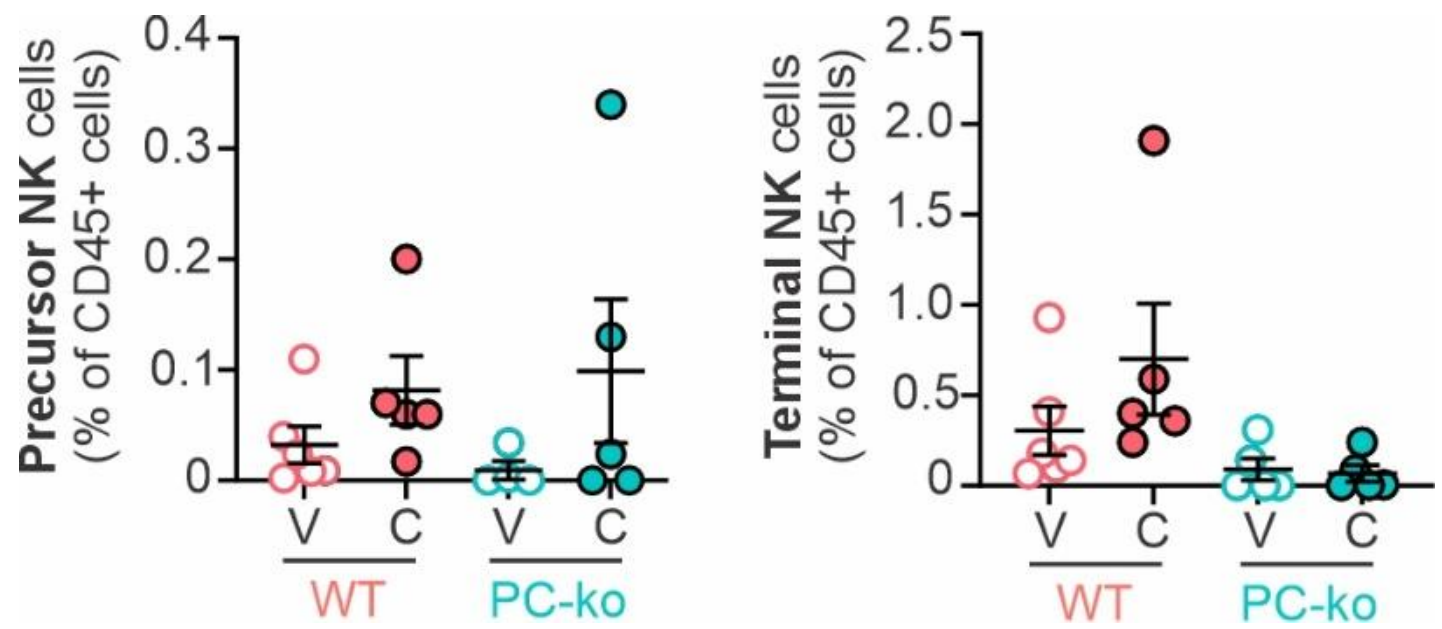

Supplementary Fig. S6: No effect of intratumoural plasma cell-deficiency on the infiltration of precursor and terminal NK cell subsets. Wild-type and PC-ko MPNSTs were generated as illustrated in Figure 3A, treated with vehicle or CDK4/6-MEK inhibition for 5-6 days, and intratumoral immune cells quantified by flow cytometric analyses. Left, Percent of precursor (early, unspecialized progenitor) NK cells. Right, percent of terminal (fully mature) NK cells. Each dot represents a tumour from an individual mouse. Error bars are SEM. Data were analyzed via one-way ANOVA with Tukey's multiple comparisons. While some trends appeared, no statistically significant differences were observed.

Supplementary Fig. S7

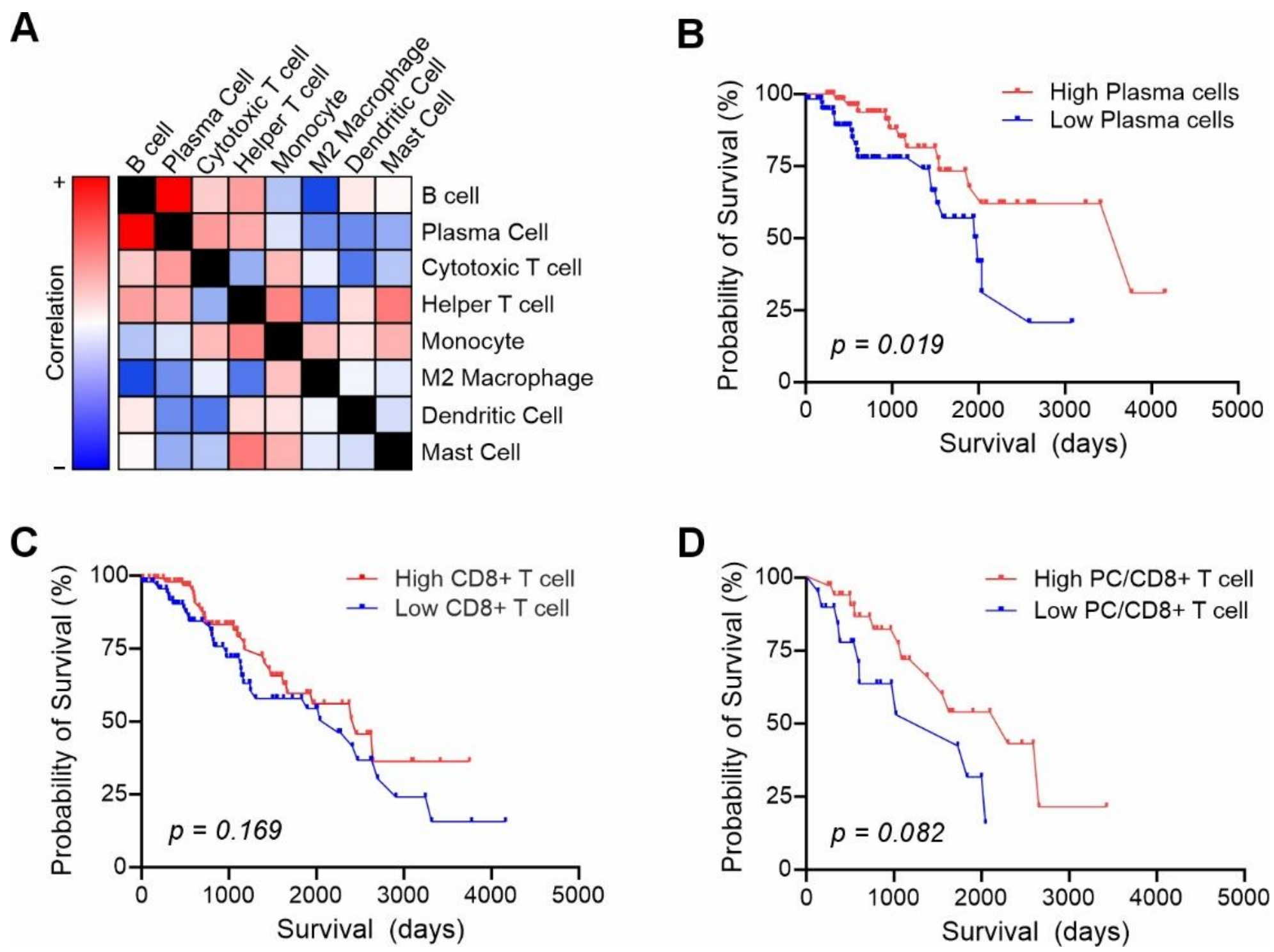

Supplementary Fig. S7: Plasma cells correlate with CD8+ T cells in patient sarcomas and predict prolonged patient survival. CIBERSORT was used to deconvolute and predict immune cell subsets from publicly available RNA sequencing data in TCGA SARC. (A) Pearson correlation matrix of immune cell subsets with each other. Red, direct correlations (r = 1); blue, indirect correlation (r = -1). Panels B-D: Kaplan Meier survival analyses based on gene signatures for (B) plasma cells, (C) T cells, and (D) both plasma cells and T cells. Any samples with null values for either immune cell signature were removed and remaining samples were bisected at the upper and lower 45th percentile. P values are indicated.

Supplementary Table S1: Gene-specific primer sequences used for quantitative RT-PCR

Gene	Forward Primer	Reverse Primer
Cd4	CTGATGTGGAAGGCAGAGAAG	GAGACCTGGGGTATCTTGAGG
Foxp3	CTCATGATAGTGCCTGTGTCCTCAA	AGGGCCAGCATAGGTGCAAG
B2m	AGACTGATACATACGCCTGCAG	CGAGGTTCAAATGAATCTTCAG
H2-k1	TCCATCCACTGTCTCCAACA	CTGGAGCCAGAGCATAGTCC
H2-d1	CATGGTGATCGTTGCTGTTC	CTGGAGCCAGAGCATAGTCC
H2-Ab1	ACCCAGCCAAGATCAAAGTGC	TGCTCCACGTGACAGGTGTAGA
H2-Aa	CTGACCACCATGCTCAGCTCT	TACTGGCCAATGTCTCCAGGAG
Igkc	GTGCCTCAGTCGTGTGCTTC	TGCTGCTCATGCTGTAGGTG
Cxcl10	GTGAGAATGAGGGCCATAGG	TTTTTGGCTAAACGCTTTCAT
Cxcl9	ATCTTCCTGGAGCAGTGTGG	AGTCCGGATCTAGGCAGGTT
Gapdh	GTTGTCTCCTGCGACTTCA	GGTGGTCCAGGGTTTCTTA

Supplementary Table S2: Antibodies used for immunofluorescent detection of intratumoral immune cells and proteins.

Antibody	Vendor and Cat No.	Dilution	Fluor
Kappa Light Chain	Abcam ab190484	1:50	647
CD8a	ThermoFisher MA1-10301	1:50 - amplified	647
CD4-eFluor 660	ThermoFisher 50-9766-82	1:10	none
MHC-I	ThermoFisher MA5011723	1:10	568
MHC-II	ThermoFisher 14-5321-82	1:100	488
PD-L1	Proteintech 66248-1-Ig	1:25	568

Supplementary Table S3: Antibodies and reagents used for flow cytometric analyses of tumour infiltrating immune cells.

Marker	Fluor	RRID
CD274	BUV395	AB_2743120
Viability	N/A	--
CD24	BUV496	AB_2870229
CXCR3	BUV563	AB_2870915
LAG-3	BUV615	AB_2875328
CD11b	BUV661	AB_2870249
CD19	BUV737	AB_2870111
CD26	BUV805	AB_2917539
CD26	BV421	AB_10895908
F4/80	eFluor 450	AB_1548747
CD279	BV480	AB_2744041
CD44	BV510	AB_2738011
CD11c	eFluor 506	AB_2637427
CD45R	BV570	AB_10900264
Ly6C	BV605	AB_2737949
CD62L	BV650	AB_2738597
CD27	Super Bright 702	AB_2722949
CD117	BV750	AB_2872100
TIM-3	BV786	AB_3684559
CD138	BB515	AB_2739597

CD45	Alexa Fluor 532	AB_11219868
CD4	RB613	AB_3686235
MHC-II	PerCP	AB_2191073
NK1.1	BB700	AB_2744491
CD103	RB705	AB_3685935
CD8	RB744	AB_3685780
CD69	RB780	AB_3626319
CTLA-4	PE	AB_10895585
CD3	RY586	AB_3684087
IgM	PE-Dazzle 594	AB_2566585
CD80	PE-Cy5	AB_468774
CD206	PE-Fire 700	AB_2922468
CD86	RY775	AB_3692685
Ly6G	PE-Fire 810	AB_2910290
TCRgd	APC	AB_1731824
Siglec-F	eFluor 660	AB_2574186
IgD	Spark NIR 685	AB_2888693
CD127	R718	AB_3674854
FCeR1a	APC-Fluor 780	AB_2573989
CD38	APC-Fire 810	AB_2890674
